# Comprehensive multi-omics analysis of pyroptosis for optimizing neoadjuvant immunotherapy in patients with gastric cancer

**DOI:** 10.7150/thno.93124

**Published:** 2024-05-05

**Authors:** Jia-Bin Wang, You-Xin Gao, Yin-Hua Ye, Qiao-Ling Zheng, Hua-You Luo, Shuan-Hu Wang, Tao Zhang, Qin-Wen Jin, Chao-Hui Zheng, Ping Li, Jian-Xian Lin, Qi-Yue Chen, Long-Long Cao, Ying-Hong Yang, Chang-Ming Huang, Jian-Wei Xie

**Affiliations:** 1 Department of Gastric Surgery, Fujian Medical University Union Hospital, Fuzhou, China.; 2 Key Laboratory of Ministry of Education of Gastrointestinal Cancer, Fujian Medical University, Fuzhou, China.; 3 Fujian Key Laboratory of Tumour Microbiology, Fujian Medical University, Fuzhou, China.; 4 Department of Pathology, Fujian Medical University Union Hospital, Fuzhou, China.; 5 Department of Gastrointestinal and Hernia Surgery, The First Affiliated Hospital of Kunming Medical University, Kunming, China.; 6 Department of Gastrointestinal Surgery, The First Affiliated Hospital of Bengbu Medical College, Bengbu, China.; 7 Department of Gastrosurgery, Liaoning Cancer Hospital & Institute, Cancer Hospital of China Medical University, Shenyang, China.; 8 Department of Gastrointestinal Surgery, The Affiliated Tumor Hospital of Guangxi Medical University, Nanning, China.; 9 Gastrointestinal Cancer Institute, Fujian Medical University, Fuzhou, 350001, China.

## Abstract

**Background:** Pyroptosis plays a crucial role in immune responses. However, the effects of pyroptosis on tumor microenvironment remodeling and immunotherapy in gastric cancer (GC) remain unclear.

**Patients and Methods:** Large-sample GEO data (GSE15459, GSE54129, and GSE62254) were used to explore the immunoregulatory roles of pyroptosis. TCGA cohort was used to elucidate multiple molecular events associated with pyroptosis, and a pyroptosis risk score (PRS) was constructed. The prognostic performance of the PRS was validated using postoperative GC samples from three public databases (n=925) and four independent Chinese medical cohorts (n=978). Single-cell sequencing and multiplex immunofluorescence were used to elucidate the immune cell infiltration landscape associated with PRS. Patients with GC who received neoadjuvant immunotherapy (n=48) and those with GC who received neoadjuvant chemotherapy (n=49) were enrolled to explore the value of PRS in neoadjuvant immunotherapy.

**Results:** GC pyroptosis participates in immune activation in the tumor microenvironment and plays a powerful role in immune regulation. PRS, composed of four pyroptosis-related differentially expressed genes (*BATF2*, *PTPRJ*, *RGS1*, and *VCAN*), is a reliable and independent biomarker for GC. PRS^low^ is associated with an activated pyroptosis pathway and greater infiltration of anti-tumor immune cells, including more effector and CD4+ T cells, and with the polarization of tumor-associated macrophages in the tumor center. Importantly, PRS^low^ marks the effectiveness of neoadjuvant immunotherapy and enables screening of GC patients with combined positive score ≥1 who benefit from neoadjuvant immunotherapy.

**Conclusion:** Our study demonstrated that pyroptosis activates immune processes in the tumor microenvironment. A low PRS correlates with enhanced infiltration of anti-tumor immune cells at the tumor site, increased pyroptotic activity, and improved patient outcomes. The constructed PRS can be used as an effective quantitative tool for pyroptosis analysis to guide more effective immunotherapeutic strategies for patients with GC.

## Introduction

In recent years, we have entered a new era of using immunotherapy to treat advanced gastric cancer (GC), and PD-1 checkpoint blockade has gradually become the first-line treatment for GC 1. Despite the advantages of this treatment over previous treatments, many patients do not respond to anti-PD-1 therapy. Combination therapies and screening of specific populations for benefits are now becoming more common means of overcoming factors such as the immune evasion of tumors. In the latest NEONIPIGA phase II clinical trial, nivolumab combined with ipilimumab neoadjuvant chemotherapy was found to be feasible and had a high pCR rate 2. The Checkmate-649 trial established the importance of immunotherapy in advanced GC, and the nivolumab plus chemotherapy group showed a significant improvement in overall survival 3. Multiple combination regimens and screening of specific patients (e.g., those with a combined positive score [CPS]≥5) have shown significant advantages for patients receiving immunotherapy 4. However, the potential audience for immunotherapy remains limited 5. It is still necessary to further explore the population that can benefit from immunotherapy, and it is crucial to clarify the key pathways that limit anti-tumor immunity.

A major contributing factor to the limited efficacy of anti-tumor immunotherapy is the paucity of tumor-infiltrating lymphocytes (TILs) in the tumor microenvironment (TME) 6. The ability to convert immune “cold” tumors into “hot” tumors, which are more amenable to immunotherapy, represents a significant breakthrough in cancer treatment 7. In recent years, inducing necroptosis and/or pyroptosis in a tumor-specific manner has profoundly impacted the tumor immune microenvironment and response to immunotherapy 8. However, the potential of pyroptosis in enhancing anti-tumor immunity in GC needs to be further explored.

Recently, pyroptosis has been widely studied as a mechanism of inflammatory cell death in cancer immunotherapy 8. Using a biorthogonal system to induce *GSDME*-mediated pyroptosis, Wang *et al.* applied the system to tumor cells and showed that pyroptosis-induced inflammation could elicit robust anti-tumor immunity and act synergistically with PD-1 inhibitors 9. Lu *et al.* designed a novel NK-tailored chimeric costimulatory switching receptor containing PD-1 to convert inhibitory PD-1/PD-L1 signals into activating signals, thereby effectively enhancing the anti-tumor activity against lung cancer cells by triggering pyroptosis 10. In addition, GSDMD in antigen-presenting cells suppressed the ability of macrophages and DCs to present tumor-associated antigens during PD-L1 inhibition and suppressed cytotoxic CD8+ T cell anti-tumor responses 11. Therefore, comprehensive studies on pyroptosis will enhance our understanding of how to develop successful immunotherapy strategies for GC in clinical settings when optimizing combination regimens.

In this study, we explored the immunomodulatory effects and multiple molecular events involved in pyroptosis and constructed a pyroptosis risk score (PRS). We validated the ability of the PRS stability to predict the prognosis of patients with GC at multiple medical centers. Single-cell sequencing and multiplex immunofluorescence were used to elucidate the immune cell infiltration landscape. The PRS^low^ is a marker of the effectiveness of immunotherapy and can be used to screen patients with GC who will benefit from neoadjuvant immunotherapy. Our study elucidated the immunoregulatory role of pyroptosis in the TME and identified the PRS as a useful quantitative tool for pyroptosis analysis to guide surgeons in selecting more effective immunotherapy strategies for patients with GC.

## Results

### The overall design of this study

**Figure 1:**
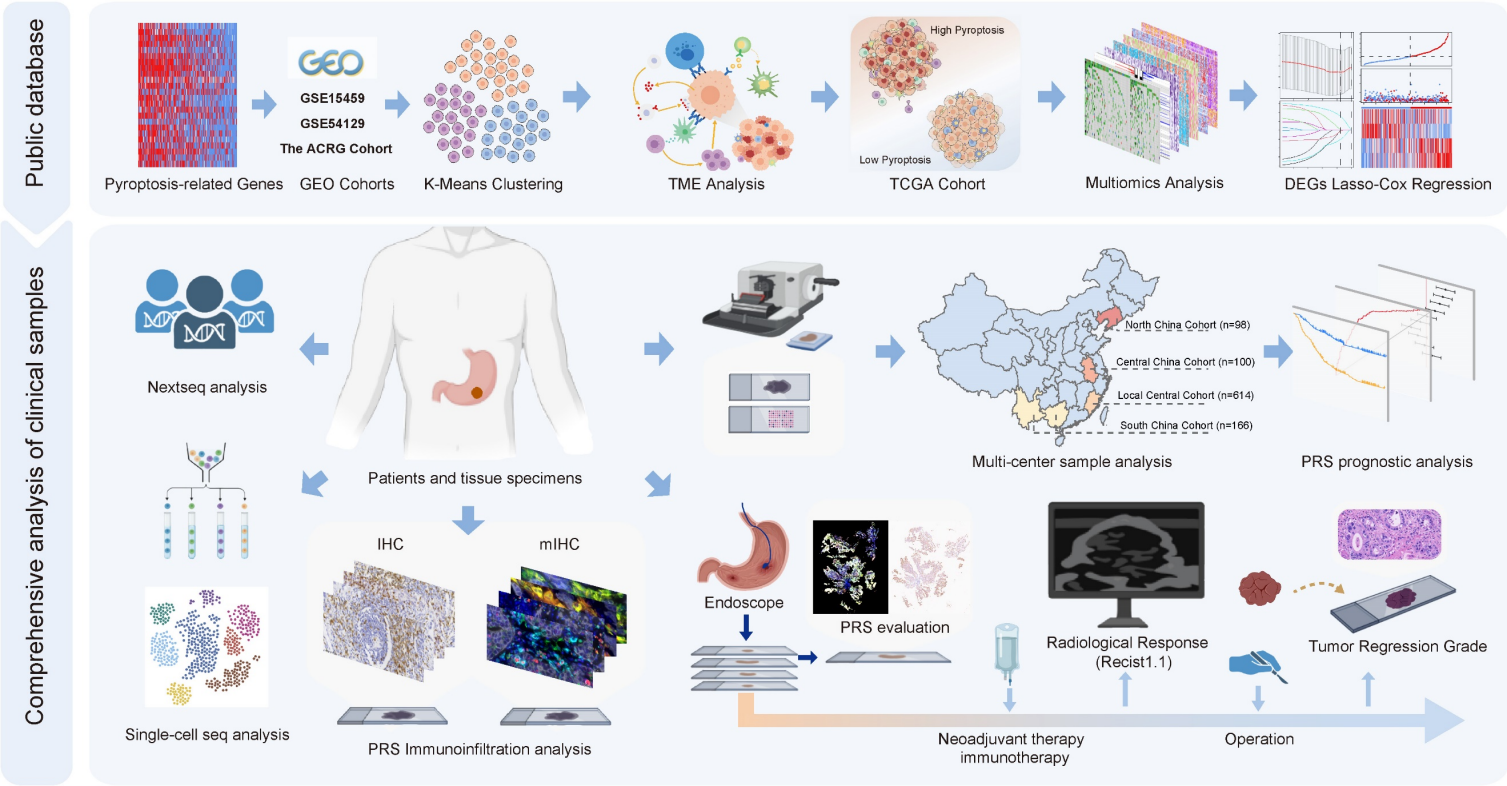
** The overall design of the study.** GC, gastric cancer; DEGs, differentially expressed genes; IHC, immunohistochemistry; mIHC, multiplex immunohistochemistry staining; PRS, pyroptosis risk score.

Figure 1 illustrates the flow chart of this study. Figure S1 illustrates the inclusion and exclusion criteria for the clinical GC patient samples. In this study, we utilized large-sample GEO data (GSE15459, GSE54129, and GSE62254) to investigate the immunoregulatory role of pyroptosis. The Cancer Genome Atlas (TCGA) cohort was used to elucidate immune regulation and multiple molecular events involved in pyroptosis, and to constructed a pyroptosis risk score (PRS). The involvement of pyroptosis and immune regulation was confirmed by analyzing transcriptome and whole-exome sequencing data obtained from our center, as reflected by the PRS. The prognostic performance of the PRS was validated using postoperative GC samples from three public databases (n = 925) and four independent Chinese medical cohorts (n = 978). Single-cell sequencing and multiplex immunofluorescence techniques were employed to elucidate the immune infiltration landscape associated with PRS. Patients with GC who received neoadjuvant immunotherapy (n = 48) and those who received neoadjuvant chemotherapy (n = 49) were enrolled to explore the predictive value of the PRS in neoadjuvant immunotherapy.

### Pyroptosis plays an immune regulation role in GC tumor microenvironment

In this exploratory study, we used a series of pyroptosis-related genes that play key roles in GC to construct a model describing the degree of pyroptosis (Figure S2-S3, see “Construction of pyroptosis regulator phenotypes” in the Methods section). We collected a large sample of multicenter transcriptome data for comprehensive evaluation (N=603 cases), and the batch effect was corrected (Figure S4A-C). Three distinct pyroptosis patterns were identified by unsupervised clustering based on pyroptosis-related genes, and the clusters were well distinguished (Figure S4D-G). Most of the pyroptosis-related genes were associated with a good prognosis in patients with GC, and these genes were significantly enriched in Cluster A, which we defined as the high-pyroptosis group, followed by Cluster B (medium-pyroptosis group) and Cluster C (low-pyroptosis group) (Figure S5A-B). Survival analysis showed that the high pyroptosis group had a good prognosis for patients with GC, whereas the low pyroptosis group had the worst prognosis (Figure S5C-D).

**Figure 2:**
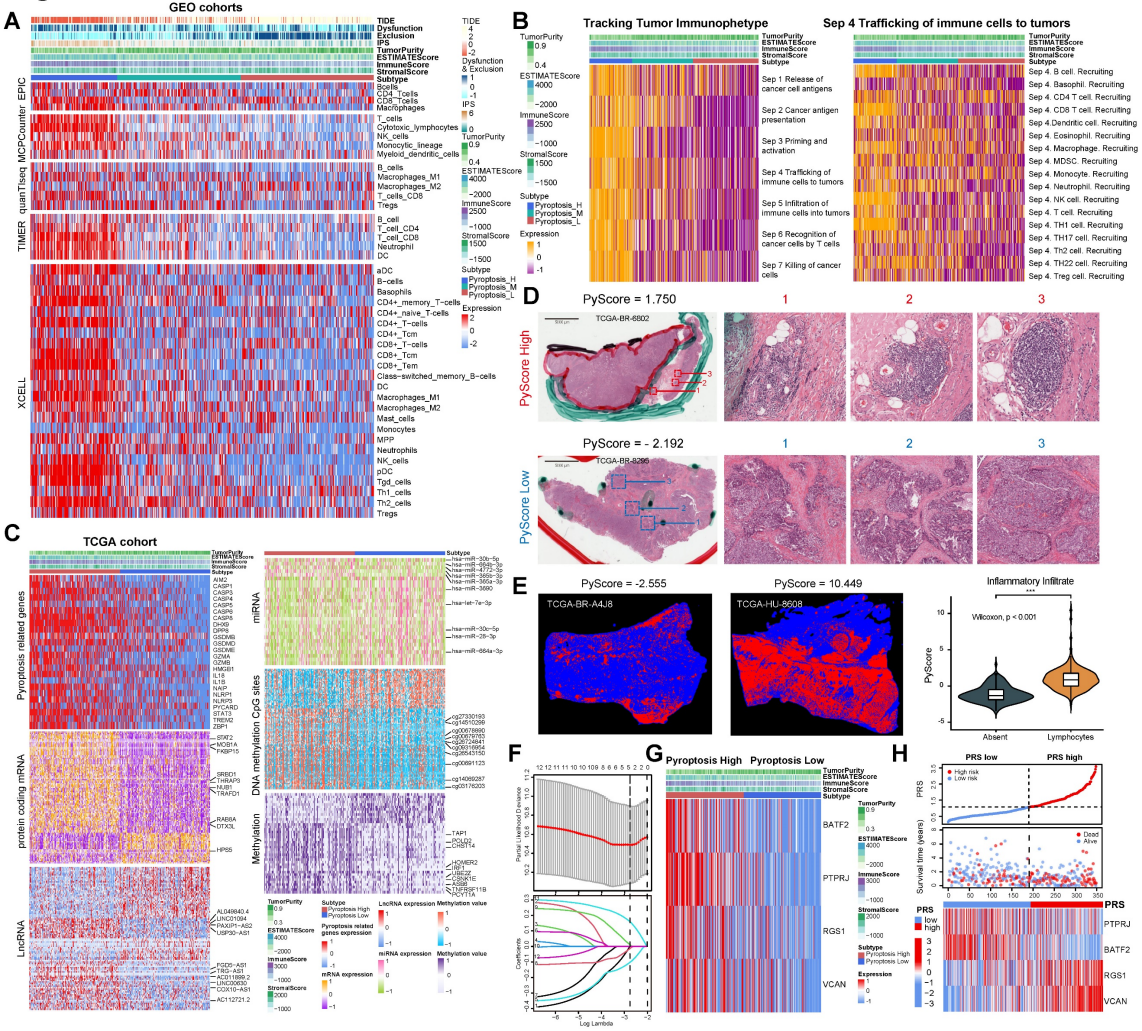
** Comprehensive analysis of gastric cancer cell pyroptosis on the characteristics of the tumor microenvironment and the establishment of pyroptosis risk score (PRS). (A)** Integrated heatmap showed the frequency and immunoscore of the tumor microenvironment (TME)-infiltrating cells in the three pyroptosis phenotypes. **(B)** The Tracking Tumor Immunophenotype (TIP) algorithm was used to visualize the anti-tumor immune status of pyroptosis using an integrated heatmap. **(C)** The comprehensive heatmap showed the differential molecular events related to different degrees of pyroptosis in TCGA-STAD, including long noncoding RNA (lncRNA), protein-coding RNA expression, microRNA (miRNA) expression, methylated CpG sites, and differentially expressed genes in methylated regions. **(D)** Representative images of pathological hematoxylin & eosin (HE) staining of high- and low-degree pyroptosis. **(E)** Deep learning was used to identify the data of tumor-infiltrating lymphocytes from the HE pathological images of The Cancer Genome Atlas stomach adenocarcinoma (TCGA-STAD). **(F)** Least absolute shrinkage and selection operator (LASSO) Cox regression were used to determine the optimal lambda and corresponding coefficients of the four indicators. **(G)** The heatmap showed the expression of four key genes in the PRS in TCGA-STAD. **(H)** The stepwise multivariate Cox proportional regression risk application model was used to obtain the risk score of each patient with gastric cancer in TCGA, and the patients were classified according to the median. *, *p* <0.05; **, *p* <0.01; ***,* p* <0.001.

Different calculation methods were used to estimate the levels of immune cell infiltration associated with different degrees of pyroptosis (Figure 2A). The results of multiple analysis showed that the high-pyroptosis group could be interpreted as immune “hot” tumors, related to the high infiltration of a variety of immune cells with anti-tumor activity, such as T, CD4^+^T, CD8^+^T, and NK cells, and were labeled as “immune activated” TIDE, IPS, ESTIMATE, and other algorithms (Figure 2A). In contrast, the low pyroptosis group was associated with an “immunosuppressive pattern” and with “low dysfunction” and “high immune rejection scores” according to the TIDE algorithm (Figure 2A). Furthermore, we identified the secretory factors in subgroups with different degrees of pyroptosis. The results revealed that the high-pyroptosis group exhibited elevated expression levels of immune checkpoint- and activation-related factors, which play important roles in immune regulation in the TME (Figure S6A). Moreover, we utilized the TIP algorithm to visualize the anti-tumor immune status during pyroptosis. The results revealed that the high-pyroptosis group exhibited a stronger anti-tumor immune state, which facilitated the recruitment of anti-tumor immune cells (Figure 2B). Gene set expression analysis (GSEA) revealed that the high pyroptosis group exhibited enriched immune inflammatory response signals and activation of the pyroptosis pathway (Figure S6B). Gene set variation analysis (GSVA) revealed that the high-pyroptosis group was significantly enriched in a series of signaling pathways related to the anti-tumor immune response (Figure S7A). Both Gene Ontology (GO) enrichment and Kyoto Encyclopedia of Genes and Genomes (KEGG) analyses revealed that the differentially expressed genes were enriched in various immune response-related pathways (Figure S7B-C). In addition, the abundance of a variety of immune-infiltrating cells increased with the degree of pyroptosis, as revealed by single-sample gene set expression analysis (ssGSEA) of immune cell markers (Figure S7D).

In summary, using multiple algorithms, we demonstrated that pyroptosis plays a key immune regulatory role in the TME of GC, indicating that pyroptosis may guide immunotherapeutic strategies in patients with GC.

### Multi-omics and immune landscape of pyroptosis

Since TCGA database provides large omics data, we continued to explore the multi-omics events associated with different levels of pyroptosis. To calculate the degree of pyroptosis in each sample, we determined the PyScore for each sample based on the mRNA expression levels of 24 pyroptosis-related genes using principal component analysis (PCA) method (orthogonal rotation) 12. (Figure S8A; see “Quantification of the degree of pyroptosis” in the Methods section for details). The PyScore strongly correlated with the degree of pyroptosis according to the expression of pyroptosis-related genes and the pyroptosis signaling pathway from the Reactome pathway database (Figure S8B-D). The PyScore was significantly different among the three pyroptosis degrees and was positively correlated with most of the pyroptosis-related genes (Figure S8E-F). Therefore, construction of the PyScore is convenient for accurately reflecting the degree of pyroptosis in each patient.

The population was divided into high- and low-pyroptosis groups according to the median PyScore. The expression of pyroptosis-related genes increased with an increase in the PyScore (Figure 2C). We identified the molecular events most likely involved in the regulation of pyroptosis, including a series of differentially expressed lncRNAs, protein-coding RNAs, miRNAs, methylation of CpG sites, and genes in methylated regions (Figure 2C). All the differential results are presented in Supplementary File 1. The top 10 molecular events with the most significant differences in each group are marked in the heatmap (Figure 2C). We found that the TTN mutation was the most frequent in the high pyroptosis group (51%), and the frequency of the TP53 mutation was the highest in the low pyroptosis group (47%) (Figure S9A-B). The tumor mutation burden (TMB) was higher in the high-pyroptosis group (Figure S9C, Figure S10A-B), and the mutant-allele tumor heterogeneity (MATH) was higher in the low-pyroptosis group (Figure S9D), indicating the susceptibility of high pyroptosis to both immunotherapy and targeted therapy. We further investigated the association between the degree of cell pyroptosis and the microsatellite instability (MSI) score, sourced from the cBioPortal database (N = 375). The results indicated no significant difference in the MSI scores between the two groups (Figure S9E). We compared the composite copy number distribution and frequency between the high- and low-pyroptosis groups by using the R package “maftools” (Figure S9F). Our analysis revealed a range of genomic changes involved in pyroptosis.

Next, we validated the immunomodulatory role of pyroptosis in the TME using TCGA data. We acquired HE staining data from the TCGA-STAD to evaluate the histopathological architecture of GC with varying degrees of pyroptosis (Figure 2D). GC tissue sections exhibiting high levels of pyroptosis (PyScore=1.750) displayed tertiary lymphoid structures. In contrast, this landscape was not observed in gastric sections with low pyroptosis (PyScore=-2.192) (Figure 2D). The number of tumor-infiltrating lymphocytes significantly increased in GC tissue sections with high pyroptosis (PyScore=10.449) (Figure 2E). The deep learning technology utilized by Joel *et al.* enabled the identification of tumor-infiltrating lymphocyte data in HE pathological images 13. The histopathological sections confirmed a higher infiltration rate of immune cells in the high pyroptosis group (Figure 2E). The extent of pyroptosis positively modulated the infiltration of multiple immune cells and exhibited a significant positive correlation with the expression of various immune checkpoints in GC (Figure S11A-B). Multiple enrichment analyses showed that pyroptosis was involved in the enrichment of multiple pathways related to immune regulation, including the MHC antigen presentation response, cytotoxicity, TCR signaling, and cell death (Figure S11C-E). These findings indicate a robust positive correlation between pyroptosis and immune cell infiltration.

To validate these results, we performed transcriptome and whole-exome sequencing of 60 GC samples obtained from our center. The PyScore was associated with the degree of pyroptosis, with higher expression levels of pyroptosis-related genes in the high-PyScore group (Figure S12A). We identified the differentially expressed lncRNAs and protein-coding RNAs between the two groups. The differentially expressed genes, which were consistent with the previous TCGA cohort, were identified and are highlighted in Figure S12A (see Supplementary File 2 for details). Multiple enrichment analysis showed that pyroptosis was enriched in a series of immune activation pathways (Figure S12B). Furthermore, whole-exome sequencing results revealed that 7q21.2, 8p23.1, and 11q13.3 exhibited a similar amplification pattern in the high pyroptosis group, as previously identified in TCGA data. A comparable trend was observed at 3q26.2 in the low pyroptosis group (Figure S12C).

In summary, our results strongly confirm the immunomodulatory role of pyroptosis in the TME and elucidate the large omics data related to pyroptosis.

### Construction of pyroptosis risk score based on pyroptosis phenotype

Given that pyroptosis is co-mediated by multi-molecular events, we next aim to explore whether it is possible to construct a convenient model for the clinical application of pyroptosis. We used a mulberry diagram to show the relationship between pyroptosis and the TCGA-STAD molecular classification (N=295) 14, TNM stage, and pathological stage (Figure S13A). Based on an analysis of the four molecular subtypes of GC, it was found that the proportion of the chromosomal instability (CIN) subtype was relatively higher in the low pyroptosis group (Figure S13B). The MSI and EBV types accounted for a relatively high proportion of the high pyroptosis group (Figure S13B). Among the four molecular subtypes, the EBV subtype showed the highest pyroptosis (Figure S13B). In addition, there was no significant correlation between the degree of pyroptosis and histological grade or TNM stage (Figure S13C). Survival analysis showed that high pyroptosis was associated with better prognosis (Figure S13D). Using the R package “pRRophetic”, we have identified that the group exhibiting a high degree of pyroptosis displays increased sensitivity to chemotherapeutic agents such as cisplatin and paclitaxel (Figure S14A). However, Submap analysis showed that only the low-pyroptosis group was significantly correlated with a lack of response to chemotherapy (Figure S14B). In addition, there was no significant difference in the benefit of postoperative chemotherapy between the two groups when the postoperative chemotherapy information was combined (Figure S14C). This indicates that the use of pyroptosis to guide clinical diagnosis and treatment remains insufficient. The observed correlation between pyroptosis and tumor immunity necessitates the development of novel clinical diagnostic and therapeutic approaches that specifically target pyroptosis.

We investigated the pivotal genes involved in pyroptosis that impact the prognosis of GC via immunity and utilized them to construct a model capable of effectively and efficiently assessing the diagnosis and treatment of GC. Therefore, key differentially expressed genes between the high- and low-pyroptosis groups were screened and then entered into the LASSO-Cox regression to generate prediction models (see “Construction of the PRS” in the Methods section for details). Ultimately, the best model included four genes (*PTPRJ, BATF2, RGS1,* and* VCAN*) that were significantly associated with prognosis and constituted the PRS (Figure 2F, Figures S15A-B). All four risk-related genes played key roles in pyroptosis (Figure 2G-H). Patients exhibiting high pyroptosis levels were partially categorized into the PRS^low^ group, whereas those with low pyroptosis levels were partially categorized into the PRS^high^ group (Figure S15C). Figure S15D elucidated the reliable predictive capability of the PRS for the prognosis of patients with GC in TCGA cohort. The PRS had a positive effect on the prognosis of patients with GC (Figure S15E-F). PCA showed that the PRS could distinguish the entire GC patient population (Figure S15G). The correlation plots demonstrated the associations among *PTPRJ, BATF2, RGS1,* and* VCAN* expression levels and the PRS (Figure S15H). We further developed a nomogram of six readily available clinical features to guide the individualized management of patients with GC (Figure S15I). Next, we comprehensively explored the clinical significance and immune landscape of the PRS in patients with GC.

### Validation of prognostic efficacy and clinical features of PRS

**Figure 3:**
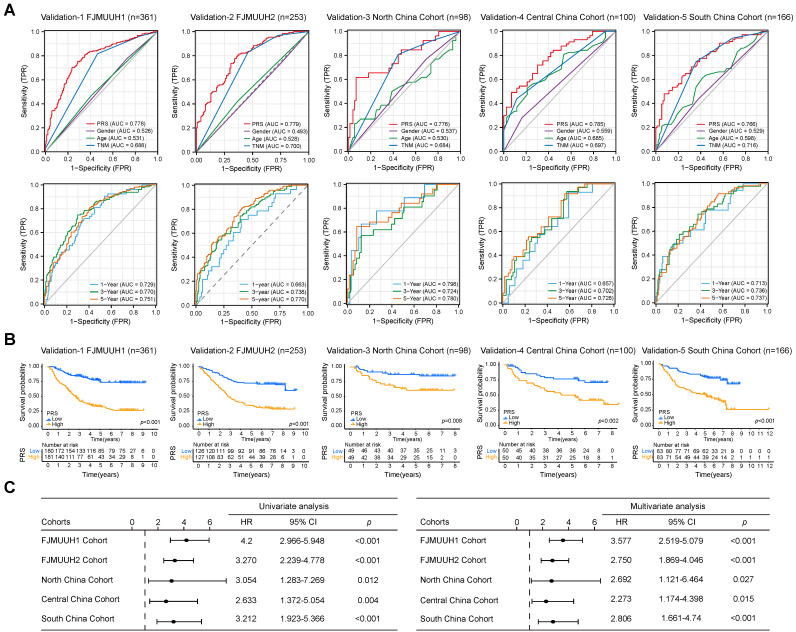
**Data from five cohorts of four independent medical centers confirmed the prognostic power of the pyroptosis risk score (PRS) in patients with gastric cancer. (A)** The diagnostic receiver operating characteristic (ROC) curve and time-related ROC curve confirmed the accuracy and stability of PRS in predicting the prognosis of patients with gastric cancer. **(B)** Kaplan-Meier curves for overall survival (OS) according to PRS in the five cohorts. **(C)** Univariate and multivariate Cox regression analyses were performed to explore the prognostic value of PRS. Variables that were statistically significant in univariate analyses were integrated into multivariate Cox regression analyses. The results of the analyses of other clinicopathological variables are shown in Tables S6-S10. *, *p* <0.05; **, *p* <0.01; ***,* p* <0.001. *P*-values for all survival analyses were calculated using the log-rank test.

To further validate the prognostic value of the PRS in GC, we explored its prognostic value in three public databases and a large number of samples from four Chinese medical cohorts. First, our analysis of the GC datasets demonstrated the ability of the PRS to effectively prognosticate patients with GC (Figure S16A). The PRS was an independent prognostic factor for patients with GC in univariate and multivariate Cox regression analyses (Figure S16B). Subsequently, we examined the prognostic potential of the PRS using immunohistochemistry (IHC) of postoperative specimens from the five medical cohorts (Figure 3 and S17, Table S1-5). They included two medical cohorts from our center, Northeast China, Central China, and Southwest China Medical Cohorts. The results showed that the diagnostic and time-dependent receiver operating characteristic (ROC) curves indicated that could PRS reliably predict prognosis in the five cohorts (Figure 3A). The prediction accuracy of PRS was significantly better than that of the AJCC8th, age, and gender (Figure 3A). Survival analysis revealed a significant survival advantage for the PRS^low^ group in all five cohorts (Figure 3B). Univariate and multivariate Cox regression analyses in multiple cohorts confirmed the prognostic value of PRS (Figure 3C, Table S6-10). The prognostic power of the PRS in each center is summarized in Table 1. Subsequently, we performed stratified analysis based on AJCC8th, differentiation, and the PRS all showed the same trend (Figure S18A-E). The lack of statistical significance in Stage I in the Northeast, Central, and Southwest China cohorts may be due to the small sample size and low mortality rates after stratification (Figure S18C-E).

Overall, multicenter data support PRS as a reliable prognostic biomarker for GC, independent of clinicopathological features.

### Deconstructing pyroptosis and the immune landscape of PRS

We performed IHC staining for key markers of pyroptosis by collecting tissue microarrays from 361 patients with postoperative GC. The expression levels of four risk-related genes (*PTPRJ, BATF2, RGS1,* and* VCAN*) in the PRS and key proteins in the classical pyroptotic pathway (*GSDMD*, *Caspase1*, *GSDME*, *Caspase3*) in GC patient tissues were evaluated (Figure S19A, B). The expression of key proteins in the pyroptosis pathway significantly increased in the PRS^low^ group, and there was a significant negative correlation between the expression of key pyroptosis proteins and PRS (Figure S20A, B). We clustered GC patients with FMUUN_RNA-Seq at our center according to different PRS levels and found that the expression levels of each pyroptotic protein were higher in the PRS^low^ group (Figure S20C). GSEA found that in the HALLMARK and KEGG pathway sets, apoptosis and immune-related pathways were enriched in the PRS^low^ group (Figure S20D-E), indicating that PRS could be used to evaluate the immune regulation of pyroptosis.

**Figure 4:**
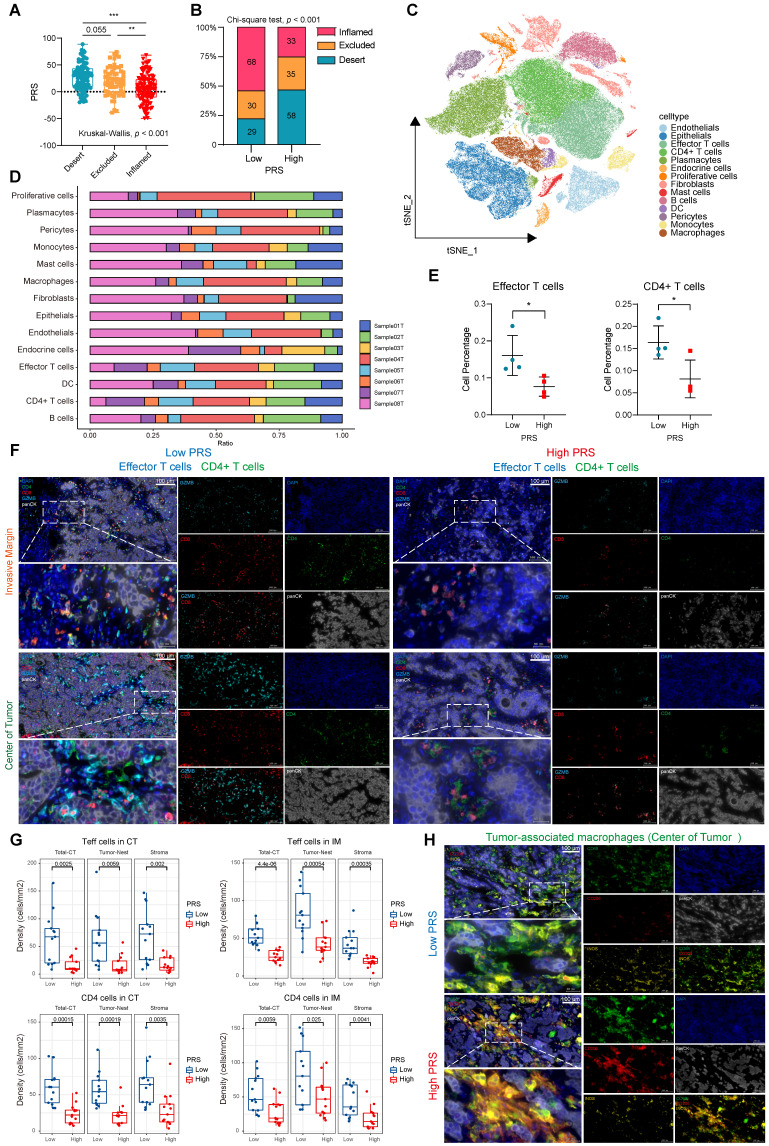
** Pyroptosis risk score (PRS)-specific landscape of the tumor immune microenvironment. (A)** The differences of PRS among the three immunophenotypes (inflamed/excluded/desert) were compared. Data are presented as the mean ± SD and were analyzed using the Kruskal-Wallis test. **(B)** The immunophenotype composition of PRS^low^ and PRS^high^ were compared. Data were analyzed using Chi-square test. **(C)** The t-SNE plot demonstrates the expression patterns of 14 specific cell clusters. **(D)** Bar graphs show the proportion of each cell cluster in eight samples, comprising four with the highest PRS (Sample01T, PRS = 74.321; Sample03T, PRS = 77.978; Sample06T, PRS = 88.530; Sample08T, PRS = 76.615) and four with the lowest PRS (Sample02T, PRS = -48.94; Sample04T, PRS = -49.564; Sample05T, PRS = -40.841; Sample07T, PRS = -42.742). **(E)** The percentage of effector and CD4^+^ T cells were compared between PRS^low^ (n = 4) and PRS^high^ (n = 4). Data are presented as the mean ± SD and were analyzed using Student's *t*-test. **(F)** Representative images show the expression of effector (GZMB^+^CD8^+^) and CD4^+^ T cells in the tumor center (CT) and invasive margin (IM) in both PRS^high^ and PRS^low^ groups on multiple immunofluorescence staining (CD8-red, GZMB-cyan, CD4-green, panCK-white, and DAPI-blue; n = 26; Scale bar = 100 μm). **(G)** Box plots show the densities of effector (Teffs) and CD4+ T cells in PRS^low^ and PRS^high^ groups, as well as their distribution at different sites in the tumor nest and stroma. (Data were analyzed using Student's *t*-test; The upper and lower ends of the box indicate the interquartile range of values. The lines in the box indicate the median and each dot signifies the corresponding value obtained from individual samples; PRS^low^: n = 13, PRS^high^: n = 13). **(H)** Representative images show the expression of tumor-associated macrophages in the tumor center (CT) in both PRS^high^ and PRS^low^ groups with multiple immunofluorescence staining (CD68-green, CD206-red, iNOS-yellow, panCK-white, and DAPI-blue; n = 26; Scale bar = 100 μm). **, p <0.05; **, p <0.01; ***, p <0.001.

To further structure the immune landscape of PRS, we performed immune cell abundance clustering of patients at our center using the ssGSEA algorithm. We divided these patients into three groups, namely “high”, “medium” and “low” immune groups (Figure S21A). IHC was used to evaluate the expression of CD8^+^ immune cells at the center of the tumor (CT) and invasive margin (IM) to verify the infiltration of immune cells in the three groups. The immune “high” group was confirmed to exhibit a pathologically “immune-inflamed” phenotype, while the immune “middle” group displayed an “immune-excluded” pathology and the immune “low” group demonstrated an “immune-desert type” phenotype (Figure S21B-C). We observed significant variations in the PRS across the three immune subtypes, with elevated scores for the immune-excluded subtype and reduced scores for the immune-inflamed subtype (Figure S21D, Figure 4A). The proportion of immune-inflamed type tumors in the PRS^low^ group was 53.5% (68 cases), which was significantly higher than the proportion of immune-inflamed tumors in the PRS^high^ group (26.2%, 33 cases) (Figure 4B). Furthermore, we quantitatively assessed several immunophenotypes (including CD45^+^ leukocytes, CD3^+^ and CD8^+^ cytotoxic T cells, CD4^+^ helper T cells, CD45RO^+^ activated and memory T cells, and FOXP3^+^ regulatory T cells; Figure S22A). The infiltrating abundance of CD4^+^, CD45^+^, CD3^+^, and CD8^+^ cells in the CT and IM groups was significantly increased in the PRS^low^ group. FOXP3^+^ cells in the PRS^high^ group were more abundant than those in the CT and IM groups, indicating an immunosuppressive state (Figure S22B). Spearman's correlation analysis of the PRS and immune cell expression showed similar trends (Figure S22C). In addition, we assessed the correlation between the PRS and the infiltration and polarization status of tumor-associated macrophages (TAMs; Figure S22D). The analysis revealed a significant positive correlation between the PRS and the infiltrating abundance of CD206^+^ cells (representing M2-type macrophages) on CT (Figure S22E). It has been suggested that PRS may reflect the spatial distribution characteristics and functions of T cells and TAMs.

In recent years, unsolved questions on tumor immunology have been elucidated with the rapid development of single-cell sequencing analysis methods. We performed scRNA-seq analysis on eight samples at our center, comprising four with the highest PRS and four with the lowest PRS. To delineate the transcriptome profile of tumor cells, we performed an unsupervised cell clustering analysis, which revealed 14 unique cell clusters (endothelials, epithelials, effector T cells, CD4^+^ T cells, plasmacytes, endocrine cells, proliferative cells, fibroblasts, mast cells, B cells, DCs, pericytes, monocytes, and macrophages; Figure 4C), each defined by marker genes (Figure S23A; see Supplementary File 3). We analyzed the expression of key PRS genes in different cell clusters (Figure S23B-F). We quantified the contents of various cell clusters in the eight samples (Figure 4D). The most significant differences in effector T cell and CD4^+^ T cell contents were observed between the PRS^high^ and PRS^low^ populations (Figure 4E). For other cell clusters, no statistically significant differences were observed between the two groups (Figure S24A). Therefore, we used CD8 and Granzyme B (*GZMB*) to characterize effector T cells as well as CD4 to identify CD4^+^ T cells. Multiplex immunofluorescence staining was performed to investigate the effects of the PRS on infiltration and spatial distribution of these cells (Figure 4F). Effector T cells and CD4^+^ T cells were highly enriched in tumor nests and stroma in both the CT and IM in the PRS^low^ group (Figure 4F-G, Figure S24B). We further explored the characteristics of TAM infiltration polarization in different PRS populations. The results showed that low PRS had greater M1 macrophage enrichment (characterized by CD68^+^iNOS^+^), whereas high PRS had a higher M2 macrophage enrichment (characterized by CD68^+^CD206^+^), as observed only on CT (Figure 4H, Figure S24C-D). This suggests that the polarization of M2-type macrophages predominates in the PRS^high^ population and accumulates in the tumor center.

Immune landscape analysis of PRS showed that low PRS levels corresponded to increased infiltration of anti-tumor immune cells, which is usually considered a beneficial signal for immunotherapy in the diagnosis and treatment of GC. Therefore, it is necessary to evaluate the benefits of immunotherapy in patients with low PRS.

### PRS predicts the benefit of neoadjuvant immunotherapy in patients with GC

**Figure 5:**
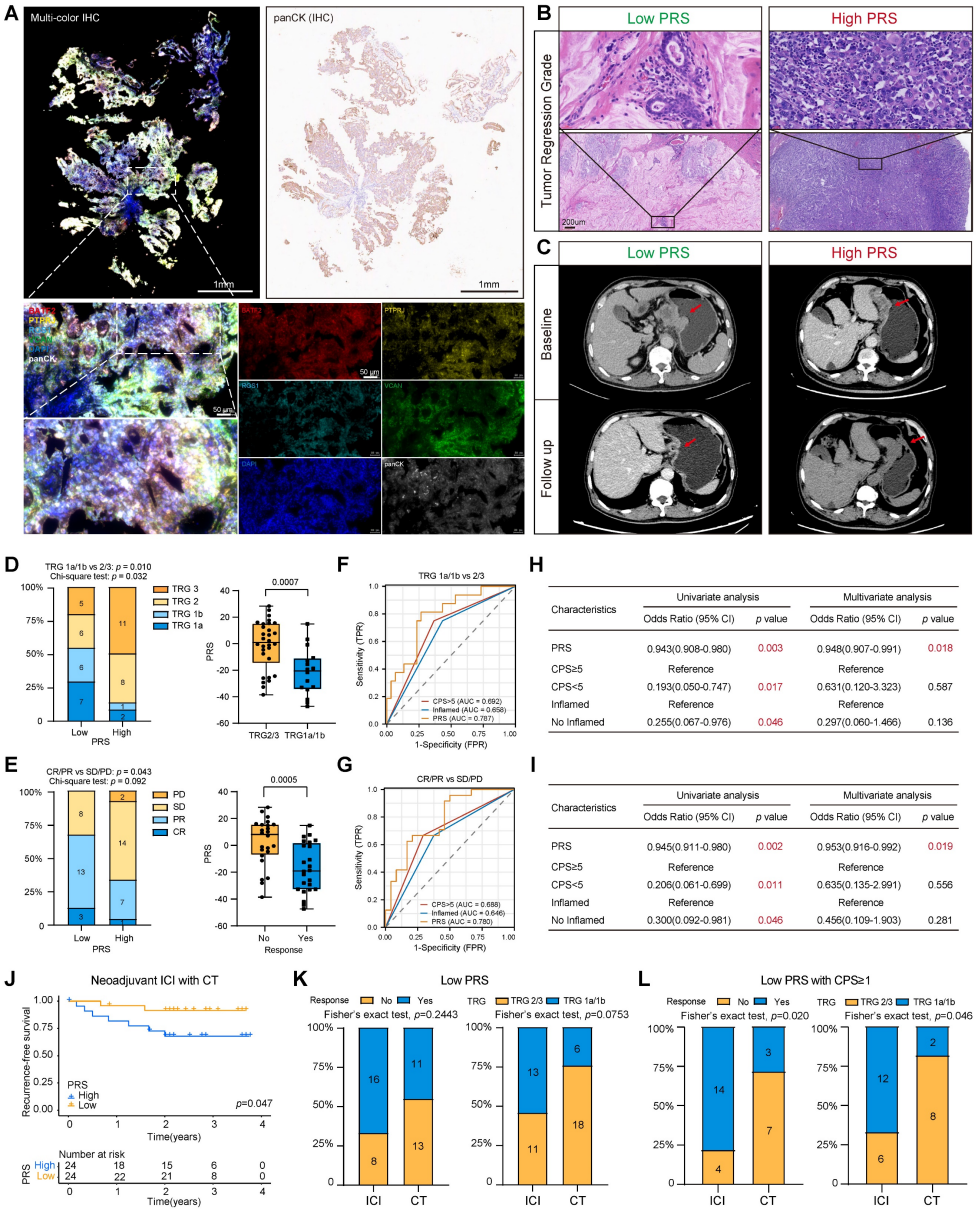
** The pyroptosis risk score (PRS) can effectively predict the treatment benefit of neoadjuvant immunotherapy in patients with gastric cancer. (A)** Typical representative images show PRS in multiple immunofluorescence staining (BATF2- red, PTPRJ- yellow, RGS1- cyan, VCAN- green, panCK- white, DAPI- blue). In addition, the white field of panCK stained by immunohistochemistry (IHC) on gastroscopic specimens is shown. Scale bar = 50 μm. **(B)** Typical representative images show the tumor regression grade of postoperative pathological tissues of PRS^low^ and PRS^high^ patients, and the CT imaging changes of PRS^low^ and PRS^high^ patients before and after neoadjuvant immunotherapy are shown in **(C)**. **(D, E)** Tumor regression grade (TRG) composition and objective response rate after neoadjuvant immunotherapy were compared between PRS^low^ and PRS^high^ patients. In addition, PRS was compared between patients who benefited from neoadjuvant immunotherapy and those who did not. Data were analyzed using Student's *t*-test. The upper and lower ends of the box indicate the interquartile range of values. The lines in the box indicate the median. **(F, G)** The receiver operating characteristic (ROC) curve was used to compare the accuracy of biomarkers (PRS, CPS, and inflammatory phenotype) in predicting response to neoadjuvant immunotherapy. **(H, I)** Univariate and multivariate logistic regression analyses were performed to confirm the predictive value of biomarkers (PRS, CPS, and inflammatory phenotype) for neoadjuvant immunotherapy (Results: TRG1a/1b). OR: odds ratio. **(J)** Kaplan-Meier survival analysis compared recurrence-free survival in PRS^low^ patients compared with PRS^high^ patients. *p*-value survival analyses were performed using the log-rank test. **(K, L)** Comparison of benefits between the group receiving neoadjuvant immunotherapy and the group receiving neoadjuvant chemotherapy alone. *, *p* <0.05; **, *p* <0.01; ***,* p* <0.001; ICI, immune checkpoint inhibitor; CT, chemotherapy.

Gastroscopic slides were collected from 48 patients with GC prior to neoadjuvant immunotherapy. Multiplex immunofluorescence staining images showed that PRS can feasibly evaluate gastroscopic tissues (Figure 5A; see “Patients and gastric tissue samples” in the Methods section for details.) Patients were assessed for tumor-regression grade (TRG) and RECIST criteria by imaging after immunotherapy (Figure 5B-C). We found greater tumor regression in the PRS^low^ group (TRG1a/1b: low PRS = 54.1%, High PRS = 12.5%, chi-square test: *p* = 0.032), and the PRS was significantly reduced in the tumor regression group (TRG1a/1b) (Figure 5D).

In addition, patients with a low PRS had a higher objective response rate, as assessed using the radiographic RECIST. Three patients with low PRS had a complete response (CR), 13 patients had a partial response (PR), and the objective response rate (ORR) was 66.7%, which was significantly better than that of patients with high PRS (ORR =33.3%) (Figure 5E). The PRS was significantly lower in the CR/PR group than in the control group (CR/PR) (Figure 5E). The ROC diagnostic curves showed that the area under the curve (AUC) of PRS was significantly better than that of CPS and the immune-inflamed phenotype (Figure 5F-G). Univariate and multivariate logistic regression analyses supported a strong association between the PRS and benefit from immunotherapy (Figure 5H-I). Moreover, patients with a low PRS had significantly better postoperative relapse-free survival than those with a higher PRS (Figure 5J). These results suggest that the PRS can be used to evaluate patients with GC who have significantly benefited from neoadjuvant immunotherapy.

The patients who received immunotherapy also received neoadjuvant chemotherapy. Therefore, 49 patients who only received the same neoadjuvant chemotherapy regimen were included as the control group. The baseline characteristics of the patients were well balanced between the two groups (Table S11). The analysis showed that patients in the PRS^low^ group had relatively better tumor regression and a higher ORR than those in the PRS^high^ group (Figure S25A-D). Recurrence-free survival did not reach statistical significance when the PRS was used to assess the benefit to patients of receiving neoadjuvant chemotherapy alone (Figure S25E). In addition, the ROC diagnostic curve of patients receiving neoadjuvant chemotherapy showed that the AUC values of PRS were 0.658 (for TRG evaluation) and 0.661 (for ORR evaluation), which are lower than the AUC values of patients receiving neoadjuvant immunotherapy (AUC=0.787 for TRG evaluation; AUC=0.780 for ORR) (Figure 5F, 5G, Figure S25F). Additionally, we confirmed the effectiveness of the PRS in predicting the response to immunotherapy among patients with GC in the PRJEB25780 cohort who received immunotherapy (Figure S25G). Overall, this trend suggests that the predictive power of the PRS may be more specific to neoadjuvant immunotherapy (Table 2, Table S12).

We aimed to determine patient groups receiving neoadjuvant chemotherapy that would benefit significantly from immunotherapy. By comparing the benefit of immunotherapy between the two groups, we found that patients in the PRS^low^ group who received neoadjuvant immunotherapy had an increased benefit compared with those who received neoadjuvant chemotherapy alone (Figure 5K). However, this phenomenon was not observed in the PRS^high^ group (Figure S25H). Recent reports on pyroptosis and tumor immunity show that pyroptosis cannot cause tumor regression in the case of the immune-desert type 9, 15, 16. Therefore, we stratified all neoadjuvant patients according to their PRS and CPS score (Figure S26A-B). Encouraging this, we found a significant benefit of immunotherapy in patients receiving neoadjuvant chemotherapy only in those with low PRS and CPS≥1 (Figure 5L). Stratified comparative analyses of the other groups showed no significant differences in these patients with or without neoadjuvant immunotherapy (Figure S26C-E).

Although stratification at our center was limited by the sample size, the results suggest that the PRS is a powerful model for evaluating the benefit of immunotherapy in patients with GC and can effectively identify patients with CPS≥1 who require neoadjuvant immunotherapy.

## Discussion

An increasing number of studies have demonstrated the pivotal role of pyroptosis in tumor immunity; however, further investigation is required to understand its anti-tumor immune function in GC. In this study, we utilized a large-scale public database to unveil the critical involvement of pyroptosis in shaping the tumor immune microenvironment, and delineated the multiple molecular events that govern the regulation of GC pyroptosis. Based on the key regulatory molecules of pyroptosis, we developed a pyroptosis risk score (PRS) that can be applied to pathological tissues.

The PRS serves as an effective quantitative tool for analyzing pyroptosis in clinical diagnosis and treatment processes, guiding the implementation of more effective immunotherapeutic strategies for patients with GC. Its ability to predict the prognosis of patients with GC is supported by large samples from multiple independent medical centers, and it can be used to identify patients eligible for neoadjuvant immunotherapy prior to surgery. PRS^low^ is associated with the activation of the pyroptosis pathway and infiltration of anti-tumor immune cells, enabling the identification of GC patients with CPS≥1 who may benefit from neoadjuvant immunotherapy.

In TME, pyroptosis can convert “cold” tumors to “hot” tumors, which enhances the likelihood of response to immunotherapy and is expected to overcome major obstacles in cancer treatment. Recent studies have demonstrated that pyroptosis in GC cells can trigger immune system activation, leading to T cell infiltration and activation 15, 16. However, the role of inflammatory release mediated by pyroptosis in cancer is complex and may promote or inhibit tumorigenesis and metastasis 17. For instance, Liu *et al.* demonstrated that pyroptosis of tumor cells can trigger cytokine release syndrome during chimeric antigen receptor (CAR) T-cell therapy and suggested that modifications to the natural CAR are necessary to decrease the likelihood of cytokine release syndrome resulting from pyroptosis 18. Researchers have shown that the induction of pyroptosis also requires the use of a combination of activators and inhibitors to release inflammatory cytokines that are harmful to the immune system 19. The release of *HMGB1*, *IL1β*, and *IL-18* mediated by pyroptosis is not well controlled, and they usually increase angiogenesis, invasion, and inhibition of cancer cell destruction by the immune system 20, 21. Therefore, cell death modalities that induce inflammation may exert disparate impacts on tumor progression and metastasis contingent upon the specific context. Additional methods that induce tumor cell pyroptosis without inducing the release of inflammatory cytokines should be screened and applied in clinical research. Given the intricate molecular mechanisms underlying pyroptosis in the immune regulation of GC and the heterogeneity of individual cancer cells, a comprehensive analysis of pyroptosis in GC is imperative.

In our study, we developed a PRS based on a comprehensive analysis of pyroptosis. Previous studies of pyroptosis in GC have elucidated the crucial role of pyroptosis in the TME through bioinformatics analysis, providing insights into predicting the prognosis of patients with GC and their response to immunotherapy 22-24. The strength of our study lies in the validation of the findings from public databases using transcriptomic and exomic data from our center. The findings of the present study emphasize the benefits of the PRS in clinical sample analysis, in contrast to previous models that were solely validated at the transcriptome level 25-27. Using large sample datasets from multiple centers in China, we demonstrated the utility of the PRS in evaluating pathological tissues and validated its efficacy in assessing the prognosis of patients with GC. Another strength of this study was the inclusion of gastric endoscopic biopsy samples from a neoadjuvant immunotherapy cohort, demonstrating the potential of PRS in guiding immunotherapy strategy for patients with GC before surgery. Considering the complex spatial structure and tumor heterogeneity of GC cells, we deconstructed immune cell regulation mediated by PRS using single-cell and multiplex immunofluorescence and found that PRS was significantly associated with the infiltration of effector and CD4 T cells. As one of the effector T cell markers in our study, *GZMB* is a serine protease that induces cell death in cytotoxic T cells. Recent studies have shown that *GZMB* cleaves *GSDME*-mediated pyroptosis in target cells, leading to enhanced anti-tumor immunity and reduced tumor growth 16. The higher abundance of *GZMB* positive cells in the PRS^low^ group may account for its capacity to effectively screen and identify patients exhibiting pyroptosis and activation of anti-tumor immune response.

The protein panel investigated in this study comprised four pivotal proteins, namely *BATF2*, *PTPRJ*, RGS1 and *VCAN*, which potentially exert direct or indirect effects on pyroptosis and immune regulation within the TME across various cell populations.* BATF2*, a protein pivotal to the PRS calculation, plays a significant role in orchestrating the maturation of dendritic cells, T cells, B cells, and various immune cells and intricately participates in crucial biological processes and signaling pathways, including inflammatory responses, tumor immunity, and tumor cell proliferation and apoptosis 28-32. The regulatory function of *BATF2* in the progression of GC was extensively elucidated in our previous study 33. Furthermore, upregulation of *PTPRJ* enhances the activation of the caspase-3-related apoptotic pathway upon stimulation with chemotherapeutic drugs, indicating the potential role of *PTPRJ* in calculating the PRS to screen chemotherapy-sensitive populations 34, 35. In addition, *RGS1*, another component of the PRS calculation, was found to be a novel marker of CD8^+^ T cell depletion, leading to sustained antigenic stimulation and T-cell depletion, according to single-cell transcriptome analysis by Bai et al 36. *RGS* is also a member of the pyroptosis-related gene model proposed by Xu *et al.*, thereby implying its pivotal role in the process of pyroptosis 37. Additionally, we observed that the association between the PRS and macrophage polarization was limited to the tumor center, possibly because of the presence of *VCAN*, a constituent molecule of the PRS. Previous studies have reported that *VCAN* can be used as a marker of monocytes and myeloid suppressor cells 38, 39. Patients with GC from the PRS^low^ group may be characterized by the infiltration of *VCAN*-mediated monocytes into the tumor center, where they form macrophages and are polarized to exert an anti-tumor phenotype. We present compelling evidence that patients with GC identified as being in the PRS^low^ group have a stronger interaction between anti-tumor immune cells and GC cell pyroptosis. These findings are of particular interest given that immunotherapy strategies typically seek to improve T cell responses to cancer.

The recent approval of immune checkpoint inhibitors has improved the therapeutic landscape of GC, ushering in a new era of immunotherapy for patients with advanced GC 40. However, the predictive value of PD-L1 expression in GC has been challenged in other clinical trials. ATTRACTION-2 showed that nivolumab was superior to placebo regardless of PD-L1 positivity and that patients with PD-L1-negative GC also benefited from immunotherapy 41. In KEYNOTE-062, despite the failure of pembrolizumab in patients with CPS ≥ 1, immunotherapy was beneficial to those with PD-L1 CPS ≥ 10 42. This suggests that the current PD-L1 and CPS statuses cannot sufficiently predict the efficacy of immune checkpoint inhibitor therapy, and the response population remains limited 43, 44. It is crucial to consider whether to base surgical decision-making and clinical practice on PD-L1 expression or CPS score when selecting patients who will benefit from immunotherapy. Based on NEONIPIGA phase II and Checkmate-649 trials, first-line immunotherapy with PD-L1 CPS≥5 is more beneficial than in those with CPS <5 2, 3. Encouragingly, PRS^low^ demonstrated the efficacy of neoadjuvant immunotherapy and was able to identify GC patients with CPS≥1 who might benefit from neoadjuvant immunotherapy. We believe that patients identified as belonging to the PRS^low^ group can undergo activation of anti-tumor immune processes through pyroptosis to expand the selection of patients who can benefit from immunotherapy. In addition, we suggest that pyroptosis, as assessed by PRS, is effective in predicting the benefit of immunotherapy in the presence of immune “fever.” *In vivo* studies in mice by Wang *et al.* showed that pyroptosis of less than 15% of tumor cells was sufficient to eliminate whole-breast tumors 9. However, critically, tumor suppression was absent in immunodeficient mice or when T cells were exhausted. Therefore, the rational use of pyroptosis in immunotherapy is crucial for GC immunotherapy and can provide better scientific grounding for clinical treatment.

In conclusion, our study demonstrated that pyroptosis activates immune processes in the TME. A low PRS correlates with enhanced infiltration of anti-tumor immune cells at the tumor site, increased pyroptotic activity, and improved patient outcomes. The constructed PRS can be used as an effective quantitative tool for pyroptosis analysis, which is applicable to pathological tissues and can guide more effective immunotherapy strategies for patients with GC.

## Methods

### Patients and gastric tissue samples

A total of 645 formalin-fixed and paraffin-embedded (FFPE) specimens were collected from Fujian Medical University Union Hospital from January 2012 to October 2015. Of these, 380 patients were used for tissue microarray. In addition, a total of 364 GC tissue and clinicopathological specimens collected between September 2008 and March 2016 from four external centers were used to test the prognostic value of PRS. Among them, 98 patients were from Liaoning Cancer Hospital and Research Institute (LCH, Shenyang, China), 100 patients were from the First Affiliated Hospital of Bengbu Medical College (BMCFAH, Bengbu, China), 60 patients were from the Affiliated Tumor Hospital of Guangxi Medical University (GMUATH, Nanning, China), and 106 from the First Affiliated Hospital of Kunming Medical University (KMUFAH, Kunming, China). Clinicopathological information about the patients with GC who were enrolled in these cohorts is provided in Tables S1 - S5. Inclusion criteria were as follows: (a) GC histological identification; (b) availability of follow-up data and clinicopathological features; (c) TNM staging of GC tumors was performed according to the 2016 Union for International Cancer Control (UICC) guidelines. Exclusion criteria were as follows: (1) non-formalin-fixed, paraffin-embedded tumor specimens at initial diagnosis, including tumor center (CT) and invasive margin (IM); (2) patients receiving preoperative chemotherapy or radiotherapy; All procedures performed in studies involving human subjects were in accordance with the Declaration of Helsinki. Regarding the gastroscopic tissue collection of patients with neoadjuvant chemotherapy, according to previous studies, gastroscopic sections with tumor area greater than 0.16 mm^2^ were well evaluated 45, and the evaluation of gastroscopic biopsy specimens and surgical specimens is highly consistent. All patients for whom tissue samples were used in this study provided written informed consent. This study was approved by the Ethics Committee of Fujian Medical University Union Hospital (Ethics Approval number: 2022KY085). All centers approved the study.

### Data collection from public databases

For RNA-seq data from The Cancer Genome Atlas stomach adenocarcinoma (TCGA), fragments per kilobase per million transcripts (FPKM) were converted to transcripts per megabase (TPM) values by the R package “limma”. For microarray data from Affymetrix arrays, we downloaded the company chip raw “CEL” file, corrected it on raw scale, and employed the multiarray averaging method through affy and simpleaffy packages to perform background adjustment and quantile normalization. Taking into account batch effects between datasets, the combatseq function in the SVA software package was used to remove batch effects in different datasets for data normalization.

### Construction of pyroptosis regulator phenotypes

To search for genes that are associated with pyroptosis and play key roles in GC, we searched the GeneCards database (www.genecards.org). To filter out pyroptosis genes, the screening criteria used were protein-coding genes that have been reported to play a key role in programmed cell death confirmed by PubMed literature search (see Table S13 for details). Subsequently, TCGA tumor (n=408) and TCGA normal and GTEx paracancerous data were collected (Figure S2), as well as RNA-seq data of cancer and paracancerous tissues from 60 pairs of postoperative resected GC samples from our center (Figure S3). These genes were found to be expressed at significantly higher levels in cancer tissues than in adjacent tissues (Figure S2, S3). Therefore, 24 pyroptosis genes that are highly likely to play a key role in the initiation and progression of GC were selected as our research subjects (*AIM2*, *CASP1*, *CASP3*, *CASP4*, *CASP5*, *CASP6*, *CASP8*, *DHX9*, *DPP8*, *GSDMB*, *GSDMD*, *DFNA5*, *GZMA*, *GZMB*, *HMGB1*, *IL18*, *IL1B*, *NAIP*, *NLRP1*, *NLRP3*, *PYCARD*, *STAT3*, *TREM2*, *ZBP1*). Then, unsupervised cluster analysis (K-means, based on Euclidean distance) was used to identify the pyroptosis phenotype of GC and to classify patients according to the expression levels of 24 pyroptosis-related molecules. Each step was performed using the R package “ConsensusClusterPlus” and the process was repeated 50 times.

### Quantification of the degree of pyroptosis

To facilitate the assessment of pyroptosis in TCGA cohort, we used the principal component analysis (PCA) (orthogonal rotation) method 12 to construct a scoring system, named PyScore, to evaluate the mRNA expression levels of 24 pyroptosis related molecules in patients with GC. As shown in Figure S8A, the variance contribution of the first four principal components were significant. Therefore, we determined that the PyScore for each patient with GC was the sum of PC1-PC4. This approach allowed for the quantification of pyroptosis in each GC sample in the TCGA cohort.

### Evaluation of immunological characteristics of the tumor microenvironment

We used CIBERSORT, EPIC, MCPcounter, QuanTIseq, TIMER, and Xcell to calculate the infiltrating abundance of immune cells in GC. To explore the signal of the pyroptosis subtype associated with the tumor immune cycle, the Tracking tumor Immuno-Phenotype algorithm (TIP) (http://biocc.hrbmu.edu.cn/TIP/index.jsp) and quantitative ssGSEA algorithm were used to measure the overall level. The tumor immune dysfunction score (TIDE) (http://tide.dfci.harvard.edu/login/) was used to predict the response of patients with GC to immunotherapy. Details of the above algorithm can be found in Table S14. The Cancer Genome Atlas stomach adenocarcinoma (TCGA-STAD) HE staining pathology is derived from the TCGA database (https://portal.gdc.cancer.gov/). Data on the use of deep learning to identify tumor-infiltrating lymphocytes from hematoxylin & eosin (HE) pathological images of TCGA-STAD were derived from the study by Joel et al 13. The results of differential analysis of protein coding mRNA, long noncoding RNA (lncRNA) and microRNA (miRNA) are detailed in Supplementary Files 1-3.

### Collection of methylation data

Methylation data (HM450K) downloaded from the GDAC database (gdac.broadinstitute.org). According to the TCGA database, CpG probes were collected in the Illumina Human Methylation 450. The probes showed “NA” values were removed. The Illumina Human Methylation 450 data of tumor and adjacent normal samples in lung adenocarcinoma were gathered at the same time by the same standards. Differentially methylation CpG sites between high pyroptosis sample and low pyroptosis sample were defined using R package “ChAMP”.

### Identification of differentially expressed RNA

The empirical Bayesian approach of R package “limma” was used to identify differentially expressed genes (DEGs) for each modification pattern. An adjusted *p-*value < 0.05 and an absolute fold change > 2 were used as the criteria for the significance of DEGs.

### Construction of the PRS

The Pyroptosis Risk Score is divided into three steps: i) TCGA GC samples were divided according to the median PyScore, and the R package “limma” was used to calculate the DEGs between high and low pyroptosis groups. Genes with adjusted *p* < 0.05 and absolute fold change > 2 were considered pyroptosis-related. ii) pyroptosis-related DEGs with *p* < 0.05 in univariate Cox regression analysis were selected. The Cox regression model with least absolute shrinkage and selection operator (LASSO) penalty was used to determine the optimal weight coefficient, and the R package “glmnet” constructed the pyroptosis risk score based on the penalized maximum likelihood estimation. This process was repeated for 1000 iterations to ensure robustness of the predicted genes and stability of the model. iii) The frequency of each model was then calculated, and the pyroptosis-related genes with non-zero regression coefficients in the best gene model (the highest frequency) were selected, and the best model was determined by 10-fold cross-validation. The pyroptosis risk score was calculated using the following formula: pyroptosis risk score =exp(i) × coef(i), where exp(i) represents the eligible gene expression and coef(i) represents the corresponding coefficient in the LASSO model. The final Pyroptosis Risk Score was calculated using the following formula:

PRS= (-0.32187)*PTPRJ+ (-0.24528)*BATF2+ 0.17219*RGS1+ 0.30769*VCAN

### Gene Set Enrichment Analysis

Gene Set enrichment analysis (GSEA) was performed using the molecular Signature database (MSigDB) to identify significantly enriched pathways among different tumor sample groups. A gene set is considered “enriched” if its enrichment score is positive, the expression level of most members of the gene set is high and the risk score is also high. Pathways with a false discovery rate (FDR) adjusted *p* < 0.05 were considered significantly enriched.

### Analysis of mutation and copy number difference

Waterfall plots of gene mutations and copy number variations in the TCGA-STAD cohort were plotted using the R package “maftools”. To analyze copy number, we used the GISTIC 2.0 definition to identify amplified genomes and missing gene sequences. Copy number gain or loss was determined by the total number of genes with altered copy number at the lesion and arm level. Using genpattern website (https://cloud.genepattern.org/) gistic2 plug-in copy number analysis, and using the hg38 human genome sequence as the reference set.

### Correlation analysis of drug sensitivity

The R package “pRRophetic” was used for prediction 46, and the “linearRidge” function in the R package “ridge” was used to construct a ridge regression model to estimate the IC_50_ of patients with GC to commonly used chemotherapy drugs.

### Immunohistochemistry and evaluation

Serial sections of FFPE samples were 4 μm in size and mounted on glass slides for IHC analysis. Sections were deparaffinized with xylene and rehydrated with alcohol. We blocked endogenous peroxidase by immersing the sections in 3% H2O2 aqueous solution for 10 min and then microwave the sections in 0.01 mol/L sodium citrate buffer, pH 6.0, for 10 min for antigen recovery. The slides were then washed with phosphate buffered saline (PBS) and incubated with 10% normal goat serum (Zhongshan Biotechnology Co., LTD., China) to eliminate nonspecific reactions. Subsequently, sections were incubated with primary antibodies overnight at 4°C. Negative controls were treated in the same way, but the primary antibody was omitted. After rinsing three times with PBS, secondary antibodies were diluted, incubated on slides for 30 min at room temperature, and stained with diamine benzidine (DAB) solution. Finally, the slides were counterstained with heme, dehydrated, and fixed with cover glass and neutral resin.

For staining of PRS, the H-score was quantified using: H- score = (1 × % weak staining)+(2 × % medium staining)+(3 × % strong staining). Immunohistochemical scoring criteria (*BATF2*, *PTPRJ*, *RGS1*, and *VCAN*) are shown in Figure S17.

To assess immune cell infiltration, five representative and independent fields were captured at ×200 magnification at the tumor center (CT) and the invasive margin (IM), as shown in Figure S21B. Next, we assisted label counting using the "Measure" plug-in in the Image-Pro Plus software to obtain the number of positive cells in the field. The average number of positive cells in the five field areas was divided by the field area (0.27mm^2^) to obtain the infiltration density of immune cells in CT and IM. The percentage/number of all positive cells is expressed as the mean of five randomly selected microscopy fields.

Inflammation, exclusion, and desert phenotypes were determined based on immunocytochemical staining slides for CD8^+^, and the three immunophenotypes were classified based on features reported in previous studies, as shown in Figure S21C.

PD-L1 expression was measured using CPS. PD-L1-positive cells included PD-L1-expressing tumor cells, lymphocytes, and macrophages. The formula was calculated as: (total number of PD-L1 positive cells/total number of viable cells) *100%. The intensity criteria for immunohistochemical staining are shown in Figure S25C.

Information and concentrations of reagents used for IHC are provided below: BATF2 (sc-293274, Santa Curz, 1:100), PTPRJ (55123-1-AP, Proteintech, 1:100), VCAN (ET7107-09, HuaBio, 1:400), RGS1 (ER64803, HuaBio, 1:200), Caspase-1 (ET1608-69, HuaBio, 1:100), Caspase-3 (ET1603-26, HuaBio, 1:100), GSDME (ER1901-12, HuaBio, 1:200), GSDMD (ER1901-37, HuaBio, 1:200), CD4 (ab183685, Abcam, 1:400), CD45 (ab10558, Abcam, 1:200), CD3 (ab16669, Abcam, 1:150), CD8 (ab4055, Abcam, 1:200), CD45RO (ab23, Abcam, 1:800), FOXP3 (ab215206, Abcam, 1:200), CD68 (ab213363, Abcam, 1:300), CD206 (ab64693, Abcam, 1:500).

The IHC results were evaluated by two independent gastroenterology pathologists who were blinded to the clinical prognosis of the patients. Approximately 90% of the scoring results were the same. When the scores of the two independent pathologists diverged, another pathologist checked the results again and selected one of the scores proposed by the first two doctors, or the three pathologists discussed the decision together.

### Multiplex immunofluorescence staining

We performed multiplex immunofluorescence staining to identify the expression of GZMB (ab209236, Abcam, 1:250), CD8 (70306S, CST, 1:100), CD4 (ab183685, Abcam, 1:200), CD68 (M0876, DAKO, 1:500), CD206 (CL488-60143, Proteintech, 1:300) and INOS (ER1706-89, HuaAn, 1:100) in GC tissues. Tumor cytokeratin was stained with CKpan. All nuclei were stained with DAPI. Briefly, formalin-fixed paraffin-embedded tissue sections were cut into 4-mm thick sections, thawed at 70 °C for 45 min, deparaffinized and fixed with formaldehyde: methanol (1:10). Then, in a pH 8.0 EDTA buffer and heat-induced antigen recovery was performed at 100% power in an 800 W standard microwave until the boiling point, and then 30% power was used for 15 min. The tissue sections were then cooled and washed in 0.02% Tris-buffered saline-Tween 20 (TBST) with gentle stirring. Then, the sections were blocked with blocking buffer (Dako, X0909) for 10 min at room temperature and then incubated with the primary antibody at 4 °C overnight. Then, the horseradish peroxidase (HRP)-conjugated secondary antibody (PerkinElmer) was incubated at room temperature for 1 h, and then the tyramide-based HRP was activated at 37 °C for 20 min. The stained signal was further amplified using Opal 540 Acetamide Signal Amplification (TSA) reagent (PerkinElmer) and incubated with TSA dilution at room temperature. Using TSA, HRP-conjugated secondary antibodies mediate the covalent binding between the Pax-5 protein and different fluorophores. After this covalent reaction, additional antigen recovery (pH 6.0 citrate buffer) was performed for 20 min to remove the bound antibody. Note: Repeat all steps in sequence for each primary antibody. Then, after counterstaining with 4′,6-diamidino-2-phenylindole (Life Technologies) at room temperature, all sections were washed five times in 0.02% TBST each for 2 min and stored in a 4 °C lightproof box C until imaging.

The Mantra System (PerkinElmer, USA) was applied to capture multispectral panoramic images after staining. The scanned slides were then analyzed by InForm software (PerkinElmer, USA) to obtain quantitative data on the region of interest (ROI). InForm can accurately count the positive cells and identify them by setting reasonable thresholds, which allows for the computation of the density and ratio of the target cells in the ROI. It also allows automated segmentation of the tumor-nest (panCK^+^) and stroma (panCK^-^) to collect quantitative data from different ROIs.

### Preparation and processing of single-cell RNA sequencing data

Single-cell gel bead emulsions were generated from single-cell suspensions using a 10×Genomics Chromium Controller. cDNA was obtained from mRNA by dribble and amplified by reverse transcription reaction according to the manufacturer's instructions. Te 10× libraries were sequenced on a NovaSeq sequencing platform (Illumina, San Diego, CA). CellRanger (version 4.0.0) was used to obtain fastq files of the raw data and annotated with the human genome reference sequence (GRCh38). Gene barcoding matrices were then obtained following the Seurat (version 4.0.4) pipeline in R software (version 4.0.5, R-Foundation, Vienna, Austria). Cells with a detected gene number below 250 or above 4000, or a high ratio of mitochondrial transcripts (more than 20%), were not included in the analysis. Following normalization and scaling, the harmony algorithm was used to remove batch effects between patients. The top 2000 highly variable genes were selected for principal component analysis (PCA) method and the top 20 principal components (PCs) were used for cluster analysis. To identify differentially expressed marker genes for each cell type, the FindAllMarkers function in Seurat was used under default parameters. Marker genes were selected as those with adjusted *p* values less than 0.05, average logFC larger than 1, and percentage of cells with expression higher than 0.25. The marker genes for cell types were as follows: epithelial cells (EPCAM, KRT5, KRT8), B cells (CD19, MS4A1, CD79A), endothelial cells (PECAM1, VWF), T cells (CD3D, CD3E), macrophages (APOE, C1QA, C1QB) and fibroblasts (DCN).

### Statistical analysis

All data were processed using SPSS 25.0 (SPSS Inc. Chicago, IL) and R software (version 4.0.0). Student's *t*-test or Wilcoxon rank-sum test was used for continuous variables. We used the χ² test or Fisher exact test to compare categorical variables of clinical characteristics. The Kaplan-Meier method was used to estimate median survival. The log-rank test was used to compare survival between two groups. The association of relevant clinicopathological variables with overall survival was assessed using the Cox proportional hazard model. Interactions between the clinicopathological parameters and responsiveness to chemotherapy were tested with the Cox model. Clustering charts based on the Z‑score normalization method were used to describe the level of the expression in each case. We defined the survival time of patients who were lost to follow‑up as the time from surgery to the last follow-up time, and the survival time of patients who were still alive at the end of the study was defined as the time from surgery to the database deadline. Two-tailed *p* values < 0.05 were indicated significant differences.

### Data availability

The data generated in this study are publicly available in the Genome Sequence Archive Database (accession numbers: HRA005558). The sequencing data generated in this study were obtained from GEO and TCGA databases. Supplementary Files 1-3 provide the source data generated in the analysis. The dataset analyzed for this study is available from the corresponding author upon reasonable request.

## Supplementary Material

Supplementary figures and tables.

Supplementary files.

## Figures and Tables

**Figure 6:**
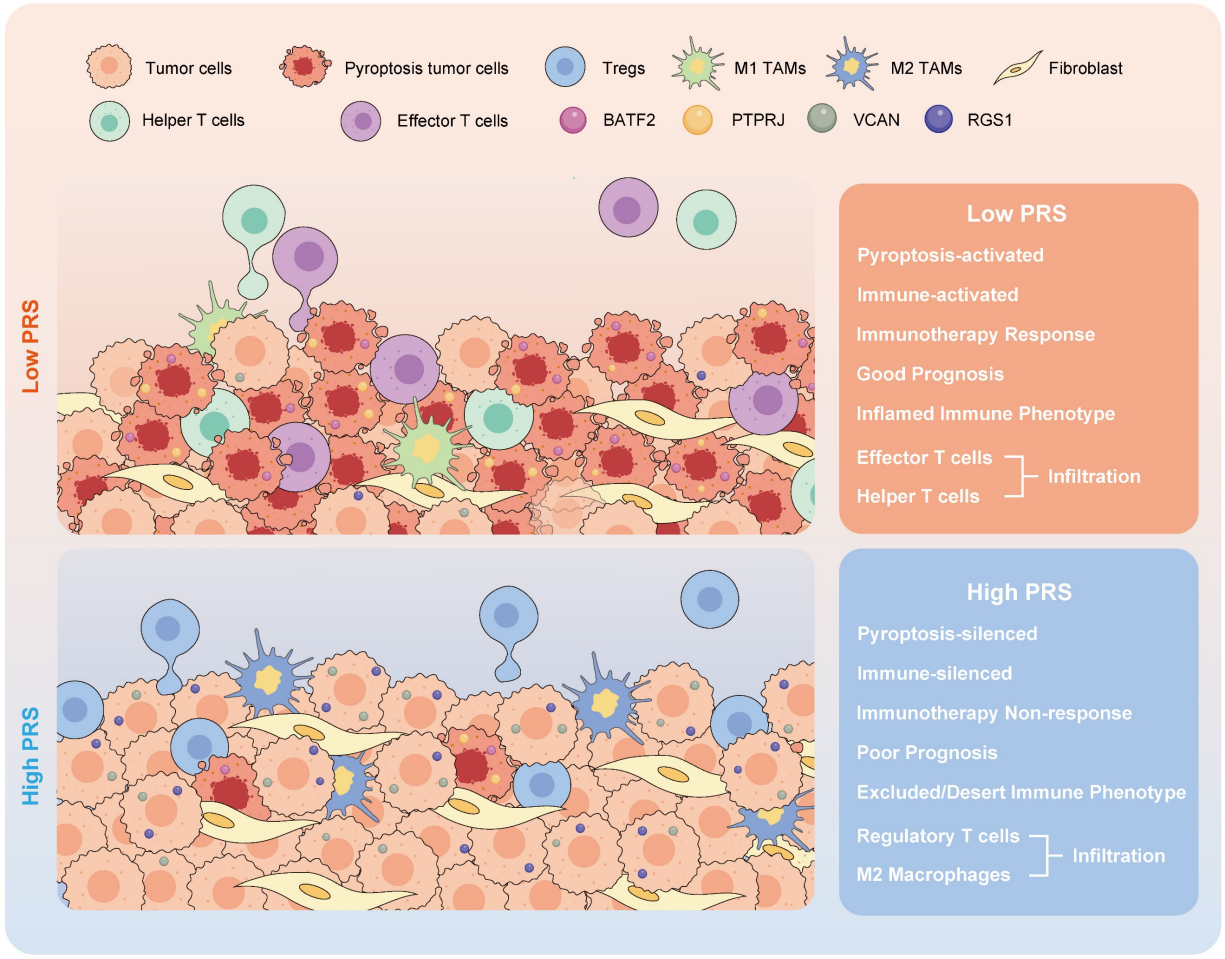
The schematic illustration depicts the attributes linked to the pyroptosis risk score (PRS) in this study.

**Table 1:** Summary of the PRS performance to predict prognosis.

	Performance to predict prognosis				
Variables	Validation-1 FJMUUH1 (n=361)	Validation-2 FJMUUH2 (n=253)	Validation-3 North China Cohort (n=98)	Validation-4 Central China Cohort (n=100)	Validation-5 South China Cohort (n=166)
**PRS**					
AUC	0.778	0.779	0.776	0.785	0.766
95%CI	0.73-0.826	0.723-0.835	0.664-0.889	0.698-0.873	0.693-0.839
Accuracy	0.731	0.719	0.847	0.68	0.479
Sensitivity	0.751	0.775	0.615	0.93	0.479
Specificity	0.714	0.661	0.931	0.491	0.926
PPV	0.698	0.704	0.762	0.58	0.829
NPV	0.765	0.739	0.87	0.903	0.704
PLR	2.623	2.289	8.862	1.828	6.499
NLR	0.348	0.34	0.413	0.142	0.563
**PRS plus AJCC**					
AUC	0.81	0.837	0.821	0.855	0.822
95%CI	0.766-0.853	0.789-0.885	0.727-0.914	0.784-0.927	0.758-0.886
Accuracy	0.74	0.767	0.755	0.77	0.777
Sensitivity	0.728	0.721	0.808	0.86	0.732
Specificity	0.75	0.815	0.736	0.702	0.811
PPV	0.719	0.802	0.525	0.685	0.743
NPV	0.758	0.737	0.914	0.87	0.802
PLR	2.911	3.887	3.061	2.885	3.865
NLR	0.363	0.343	0.261	0.199	0.33

**Table 2:** Clinicopathological Characteristics of the Neoadjuvant ICI Therapy Combined with Chemotherapy in Patients.

Variables	Total	PRS			
		Low	High	*χ*2	*P*
**Response**				4.083	**0.043**
CR/PR	24	16	8		
SD/PD	24	8	16		
TRG				6.621	**0.010**
1a/1b	16	13	3		
2/3	30	11	19		
**ypT Stage**				2.281	0.131
T0/T1	14	10	4		
T2/T3	31	13	18		
**ypN Stage**				0.019	0.889
N0	21	13	11		
N1	24	10	11		
**pTNM stage**				2.989	0.084
pCR/I	17	12	5		
II/III	28	11	17		

*P* < 0.05 marked in bold font shows statistical significance.
