# Supplementary material for: Comprehensive multi-omics analysis of pyroptosis for optimizing neoadjuvant immunotherapy in patients with gastric cancer
Source: Theranostics. 2024 May 5;14(7):2915–33. doi: 10.7150/thno.93124 (PMC11103507; doi:10.7150/thno.93124)
Supplement: Supplementary file 1 — Supplementary figures and tables. [file thnov14p2915s1.zip › Supplementary figures and tables/Figure S5.pdf]

Figure S5

A

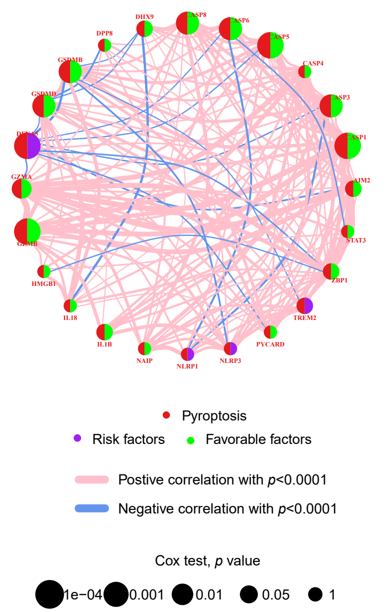

B

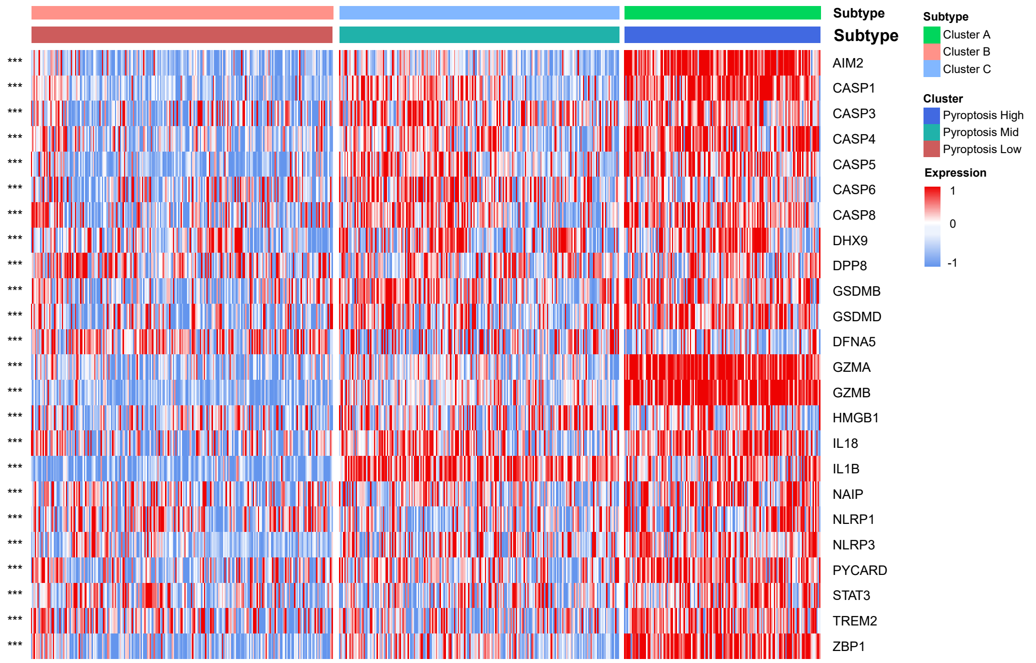

C

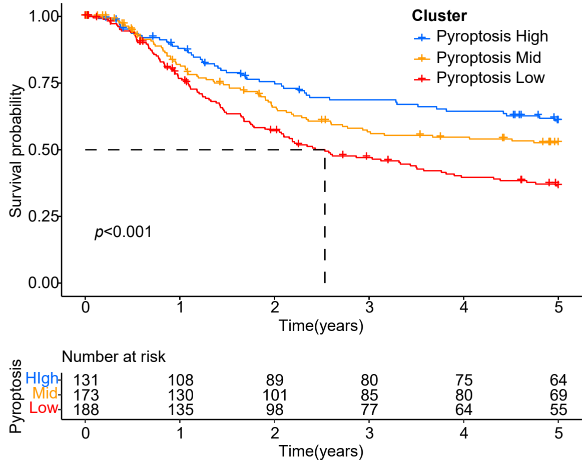

D

Variable		Cox Regression Analysis			
		Univariate		Multivariate	
		HR(95%CI)	*p*	HR(95%CI)	*p*
Gender	Female	Ref		Ref	
	Male	1.377 (0.810-1.056)	0.686	Not included	
TNM Stage	I/II	Ref		Ref	
	III/IV	4.299 (3.137-5.892)	**<0.001**	4.281 (3.119-5.877)	**<0.001**
Cluster	Pyroptosis L	Ref		Ref	
	Pyroptosis M	0.550 (0.398-0.761)	**<0.001**	0.557 (0.403-0.771)	**<0.001**
	Pyroptosis H	0.671 (0.503-0.895)	**0.007**	0.766 (0.574-1.022)	0.070
