# Supplementary material for: Comprehensive multi-omics analysis of pyroptosis for optimizing neoadjuvant immunotherapy in patients with gastric cancer
Source: Theranostics. 2024 May 5;14(7):2915–33. doi: 10.7150/thno.93124 (PMC11103507; doi:10.7150/thno.93124)
Supplement: Supplementary file 1 — Supplementary figures and tables. [file thnov14p2915s1.zip › Supplementary figures and tables/Figure S6.pdf]

Figure S6

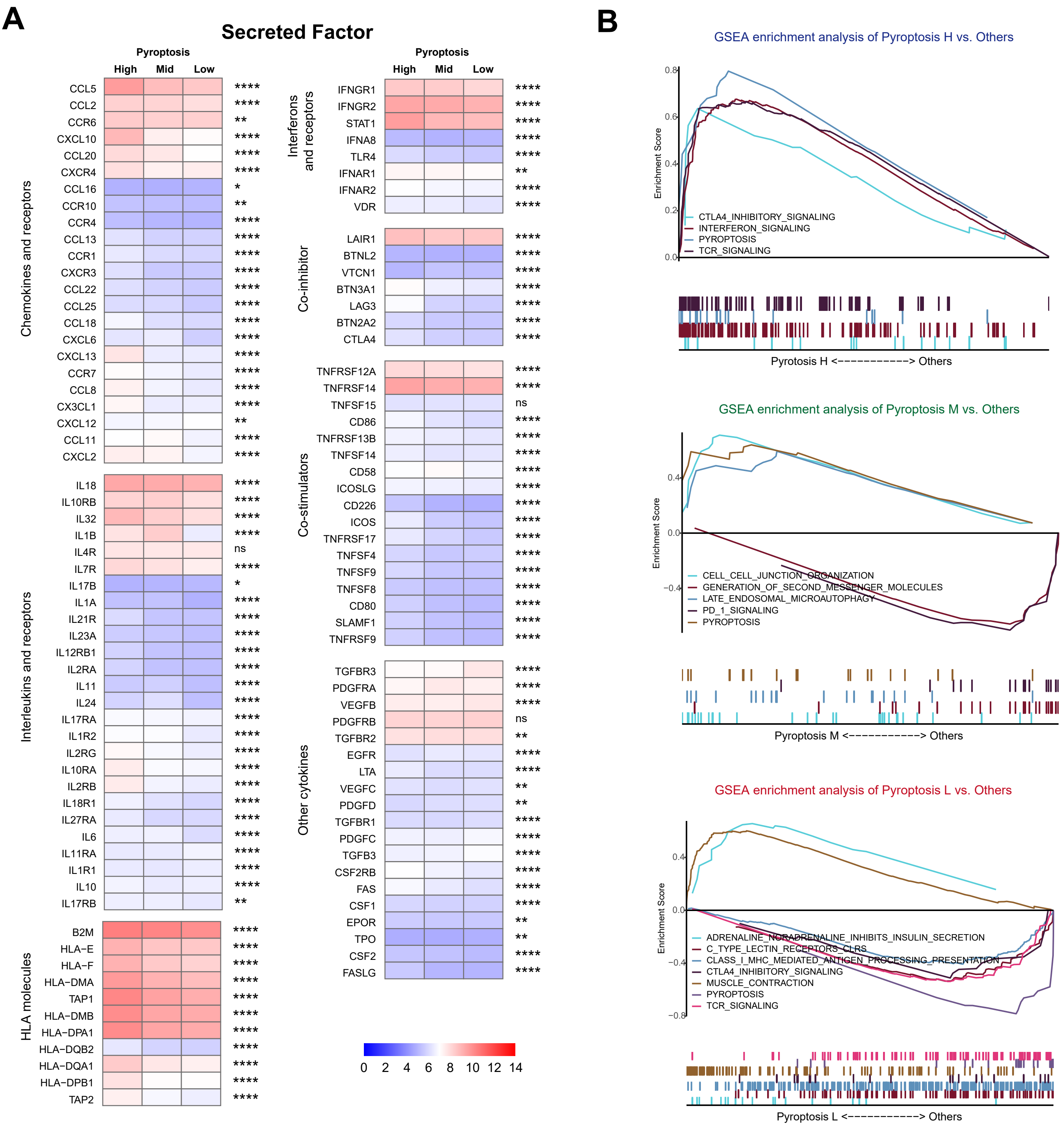

A
Secreted Factor
Pyroptosis
High Mid Low
Chemokines and receptors
CCL5 ****
CCL2 ****
CCR6 **
CXCL10 ****
CCL20 ****
CXCR4 ****
CCL16 *
CCR10 **
CCR4 ****
CCL13 ****
CCR1 ****
CXCR3 ****
CCL22 ****
CCL25 ****
CCL18 ****
CXCL6 ****
CXCL13 ****
CCR7 ****
CCL8 ****
CX3CL1 ****
CXCL12 **
CCL11 ****
CXCL2 ****
Interleukins and receptors
IL18 ****
IL10RB ****
IL32 ****
IL1B ****
IL4R ns
IL7R ****
IL17B *
IL1A ****
IL21R ****
IL23A ****
IL12RB1 ****
IL2RA ****
IL11 ****
IL24 ****
IL17RA ****
IL1R2 ****
IL2RG ****
IL10RA ****
IL2RB ****
IL18R1 ****
IL27RA ****
IL6 ****
IL11RA ****
IL1R1 ****
IL10 ****
IL17RB **
HLA molecules
B2M ****
HLA−E ****
HLA−F ****
HLA−DMA ****
TAP1 ****
HLA−DMB ****
HLA−DPA1 ****
HLA−DQB2 ****
HLA−DQA1 ****
HLA−DPB1 ****
TAP2 ****
Interferons and receptors
IFNGR1 ****
IFNGR2 ****
STAT1 ****
IFNA8 ****
TLR4 ****
IFNAR1 **
IFNAR2 ****
VDR ****
Co-inhibitor
LAIR1 ****
BTNL2 ****
VTCN1 ****
BTN3A1 ****
LAG3 ****
BTN2A2 ****
CTLA4 ****
Co-stimulators
TNFRSF12A ****
TNFRSF14 ****
TNFSF15 ns
CD86 ****
TNFRSF13B ****
TNFSF14 ****
CD58 ****
ICOSLG ****
CD226 ****
ICOS ****
TNFRSF17 ****
TNFSF4 ****
TNFSF9 ****
TNFSF8 ****
CD80 ****
SLAMF1 ****
TNFRSF9 ****
Other cytokines
TGFBR3 ****
PDGFRA ****
VEGFB ****
PDGFRB ns
TGFBR2 **
EGFR ****
LTA ****
VEGFC **
PDGFD **
TGFBR1 ****
PDGFC ****
TGFB3 ****
CSF2RB ****
FAS ****
CSF1 ****
EPOR **
TPO **
CSF2 ****
FASLG ****
0 2 4 6 8 10 12 14
B
GSEA enrichment analysis of Pyroptosis H vs. Others
Enrichment Score
0.8
0.6
0.4
0.2
0.0
CTLA4_INHIBITORY_SIGNALING
INTERFERON_SIGNALING
PYROPTOSIS
TCR_SIGNALING
Pyrotosis H <-----------> Others
GSEA enrichment analysis of Pyroptosis M vs. Others
Enrichment Score
0.4
0.0
−0.4
CELL_CELL_JUNCTION_ORGANIZATION
GENERATION_OF_SECOND_MESSENGER_MOLECULES
LATE_ENDOSOMAL_MICROAUTOPHAGY
PD_1_SIGNALING
PYROPTOSIS
Pyroptosis M <-----------> Others
GSEA enrichment analysis of Pyroptosis L vs. Others
Enrichment Score
0.4
0.0
−0.4
−0.8
ADRENALINE_NORADRENALINE_INHIBITS_INSULIN_SECRETION
C_TYPE_LECTIN_RECEPTORS_CLRS
CLASS_I_MHC_MEDIATED_ANTIGEN_PROCESSING_PRESENTATION
CTLA4_INHIBITORY_SIGNALING
MUSCLE_CONTRACTION
PYROPTOSIS
TCR_SIGNALING
Pyroptosis L <-----------> Others
