# Supplementary material for: Comprehensive multi-omics analysis of pyroptosis for optimizing neoadjuvant immunotherapy in patients with gastric cancer
Source: Theranostics. 2024 May 5;14(7):2915–33. doi: 10.7150/thno.93124 (PMC11103507; doi:10.7150/thno.93124)
Supplement: Supplementary file 1 — Supplementary figures and tables. [file thnov14p2915s1.zip › Supplementary figures and tables/Supplementary Figure Legends.docx]

**Supplementary Figure Legends**

**Figure S1. Flow chart of inclusion and exclusion of gastric cancer clinical samples in this study.**

**Figure S2.** **Transcriptomic analysis of pyroptosis-related gene expression in 60 pairs of gastric cancer and adjacent tissues collected from the TCGA and GTEx databases.** Data were analyzed using Student’s *t*-test. The upper and lower ends of the box indicate the interquartile range of values. The lines within each box indicate the median.

**Figure S3.** **Transcriptomic analysis of pyroptosis-related gene expression in 60 pairs of gastric cancer and adjacent tissues collected from our center.** Data were presented as the mean ± SD and were analyzed using Student’s *t*-test.

**Figure S4. Analysis of multicenter transcriptome data and unsupervised clustering of pyroptosis-related genes identified three distinct patterns of pyroptosis.** (A-B) Principal component analysis (PCA) shows the distribution of gene expression in three gastric cancer cohort samples (three Gene Expression Omnibus datasets) before and after correction for batch effects in (A) and (B). (C) The box chart shows that after quality control and standardization, the data of effective samples were generally on a horizontal line. (D-G) Cluster stability was evaluated using the R package “ConsensusClusterPlus” and the optimal number of clusters was determined to be three.

**Figure S5.** **Enrichment of pyroptosis-related genes in three distinct pyroptosis patterns and their impact on the prognosis of patients with gastric cancer.** (A) The line connecting two cells represents the interaction between two factors. Pink and light blue indicate positive and negative correlations, respectively. The size of the circles represents the effect of immune cells on gastric cancer survival, with purple and green indicating risk and favorable factors. (B) The composite heatmap showed the expression differences of 24 pyroptosis-related molecules in the three clusters. ***, *p* <0.001. (C) Kaplan–Meier survival analysis showed the prognosis of three groups of patients with gastric cancer with three different degrees of pyroptosis. *p*-value survival analyses were calculated using the log-rank test. (D) Univariate and multivariate Cox regression analyses were used to analyze the effects of three different degrees of pyroptosis phenotype on overall survival.

**Figure S6. The** **high-pyroptosis group exhibited a greater abundance of secreted factors and heightened activation of immune inflammatory response signals.** (A) The heatmap shows the variation in mRNA expression of chemokines and receptors, interleukins and receptors, HLA molecules, interferons and receptors, co-stimulators, co-inhibitors, and other cytokines among the three different degrees of pyroptosis (Kruskal–Wallis test). ***, *p* < 0.001. (B) Comprehensive gene set enrichment analysis showed that enriched pathways were involved in tumors with different degrees of pyroptosis.

**Figure S7. Gene set expression analysis revealed a significant enrichment of cells and signaling pathways in the high-pyroptosis group.** (A) GSVA analysis showed the activation status of biological pathways among three different degrees of pyroptosis. Heatmaps were used to visualize these biological processes, with red representing activation and blue representing inhibition. (B-C) Gene Ontology enrichment and Kyoto Encyclopedia of Genes and Genomes enrichment analysis showed the activation status of biological pathways among three different degrees of pyroptosis. (D) The Kruskal–Wallis test was used to evaluate the tumor microenvironment-related scores for three different degrees of pyroptosis. The upper and lower ends of the box indicate the interquartile range of values. The lines in the box indicate the median and the dots indicate outliers.

**Figure S8. The principal component analysis (PCA) method was employed to construct a scoring system, PyScore, and validate its ability to quantify the degree of pyroptosis in each gastric cancer sample.** (A) As can be seen from the scree plot, the variance contribution rate of the first four principal components is significant. (B) The integrated heatmap shows the abundance of the REACTOME_PYROPTOSIS pathway (gene set expression analysis [GSEA] algorithm), CLUSTER_PYROPTOSIS (representing the comprehensive expression of 24 pyroptosis-related genes), and PyScore (derived from principal component analysis [PCA]) in GC sample. (C-D) Scatter plot shows the correlation between REACTOME_PYROPTOSIS pathway (GSEA algorithm), CLUSTER_PYROPTOSIS (representing the comprehensive expression of 24 pyroptosis-related genes), and PyScore (derived from PCA analysis) in each GC sample. (E) Violin plot shows differences in PyScore in three different degrees of pyroptosis in GEO database. (F) The comprehensive heat map in the upper right corner shows the person correlation coefficient between PyScore and each pyroptosis-related gene in TCGA cohort, and the scatter plot in the lower left corner shows the pearson correlation between PyScore and each pyroptosis-related gene.

**Figure S9. The TCGA cohort's multi-omics data unveiled a multitude of genomic alterations linked to pyroptosis.** (A) Top 20 most frequently mutated genes in high- and low-pyroptosis groups. Mutated genes with a significant difference in mutation frequency between the two groups are marked in red. (B) Differences in mutation frequency between groups with high and low levels of pyroptosis. The mutation frequencies between the two groups were as follows: *TP53*: 35% in high-pyroptosis vs. 47% in low-pyroptosis groups; *DOCK3*: 15% in high-pyroptosis vs. 4% in low-pyroptosis groups; *MUC16*: 24% in high-pyroptosis vs. 37% in low-pyroptosis groups; *PIK3CA*: 9% in high-pyroptosis vs. 21% in low-pyroptosis groups; *PCDH10*: 5% in high-pyroptosis vs. 15% in low-pyroptosis groups; *CSMD2*: 3% in high-pyroptosis vs. 11% in low-pyroptosis groups; *TCHH*: 1% in high-pyroptosis vs. % 14 in low-pyroptosis groups; *ARID1A*: 14% in high-pyroptosis vs. 32% in low-pyroptosis groups; *SYNE2*: 7% in high-pyroptosis vs. 16% in low-pyroptosis groups; *AHNAK2*: 8% in high-pyroptosis vs. 18% in low-pyroptosis groups. (C-E) Tumor mutation burden (TMB), mutant-allele tumor heterogeneity (MATH), and microsatellite instability (MSI) scores were compared between high- and low-pyroptosis groups. The upper and lower ends of the box indicate the interquartile range of values. The lines in the box indicate the median and the dots indicate outliers. Data were analyzed using Student’s *t*-test. N = 375. (F) The plot illustrates the copy number GISTIC score of the gain (dark red) and loss (dark blue) of each gene in each cluster. Copy number profiles for each cluster, with gains in dark red and losses in midnight blue. Gene segments are placed according to their location in chromosomes, ranging from chromosomes 1 to 22. Copy number amplification: In high-pyroptosis cases, multiple chromosomal regions, such as 1p36.22, 7q21.2, 8p23.1, 11q13.3, and 12p13.2, exhibited significant copy number amplifications. In low-pyroptosis cases, distinct chromosomal locations, including 2p24.1, 3q26.2, 7q22.1, 9p13.3, and 12p13.1, displayed differences in the pattern of copy number amplification. Copy number deletion: In high-pyroptosis cases, deletion events were observed at various chromosomal positions, such as 2q37, 4q35.1, and 9p24.2. For low-pyroptosis cases, deletion events were observed at different chromosomal locations, such as 16p13.3, 17q24.3, and 18q21.2. Both high- and low-pyroptosis groups exhibited common chromosomal regions with copy number amplifications and deletions. For instance, regions such as 1q21.3, 6p21.1 and 19q12 were marked for amplification, while 3p21.1, 6q25.3, and 16q23.1 showed deletions in both groups.

**Figure S10. Plots illustrate the summary of tumor mutation data based on the maftools package and visualization for low-pyroptosis (A) and high-pyroptosis (B) groups.**

**Figure S11. Multiple enrichment analysis showed that pyroptosis was involved in the enrichment of multiple pathways related to immune regulation.** (A) The Kruskal–Wallis test was used to evaluate the TME-related scores for low- and high pyroptosis groups. The upper and lower ends of the box indicate the interquartile range of values. The lines in the box indicate the median and the dots indicate outliers. (B) The heatmap showed the correlation between PyScore and immune cells. Red and blue indicate positive and negative correlations, asterisk indicates *p* values. * *p* < 0.05, ** *p* < 0.01, *** *p* < 0.001. (C) GSVA showed the activation status of biological pathways between high and low pyroptosis. Heatmaps were used to visualize these biological processes, with red representing activation and blue representing inhibition. (D-E) Comprehensive gene set enrichment analysis revealed pathways enriched in low and high pyroptosis.

**Figure S12. Transcriptome and whole-exome sequencing of 60 gastric cancer samples in our center revealed multi-omics events involved in the regulation of pyroptosis.** (A) Integrated heatmap showed the frequency and immunoscore of TME-infiltrating cells and differential molecular events related to different degrees of pyroptosis in the FMUUN_RNA-Seq cohort, including long noncoding RNA and protein-coding RNA expression. (B) Comprehensive gene set enrichment analysis revealed pathways enriched in low- and high-pyroptosis groups. (C) The plot illustrates the copy number GISTIC score of the gain (dark red) and loss (dark blue) of each gene in each cluster. Copy number profiles for each cluster, with gains in dark red and losses in midnight blue. Gene segments are placed according to their location in chromosomes, ranging from chromosomes 1 to 22.

**Figure S13. Association between pyroptosis and TCGA-STAD molecular classification, TNM stage, pathological stage, and prognosis.** (A) The Sankey chart shows the correlation between pyroptosis and the clinicopathological classification of patients with gastric cancer (GC). (B) The plot illustrates the composition of four The Cancer Gene Atlas GC molecular subtypes in high- and low-pyroptosis groups. Data were analyzed using Chi-square test. N = 295. (C) The plot illustrates the composition of clinicopathological features of GC patients with high and low pyroptosis. (D) Kaplan–Meier survival analysis showed the prognosis of GC patients with high and low pyroptosis.

**Figure S14. Association of pyroptosis with chemotherapeutic drug sensitivity and chemotherapy benefit in patients with gastric cancer.** (A) The IC_50_ of The Cancer Genome Atlas patients with gastric cancer to commonly used chemotherapy drugs was estimated using the R package “prophetic”. The upper and lower ends of the box indicate the interquartile range of values. The lines in the box indicate the median and the dots indicate outliers. (B) The submap algorithm predicted the probability of response to chemotherapy in the high and low pyroptosis groups. (C) Kaplan–Meier survival analysis showed the prognosis of gastric cancer patients with high and low pyroptosis with or without chemotherapy.

**Figure S15. Establishment of pyroptosis risk score.** (A) Forest plots show hazard ratios from univariate Cox regression for the four genes and the pyroptosis risk score (PRS). (B) The forest plot shows the coefficients of four genes in PRS for predicting prognosis. (C) The Sankey plot shows the correspondence between high and low pyroptosis groups and PRS in The Cancer Genome Atlas (TCGA) cohort. (D) Kaplan–Meier survival analysis showed the prognosis of PRS^low^ and PRS^high^ patients with gastric cancer (GC). (E, F) Time-related receiver operating characteristic (ROC) curve and calibration curve confirming the accuracy of the PRS at predicting the prognosis of patients with GC in TCGA. (G) PCA showed that the PRS could distinguish the entire GC patient population. (H) The correlation plots demonstrated the associations among *PTPRJ, BATF2, RGS1, VCAN* expression levels and the PRS. (I) A composite score was constructed using nomogram Cox regression based on the PRS, gender, age, and TNM stage in the TCGA cohort.

**Figure S16. Data from GSE15459, GSE62254, and GSE84437 cohorts confirmed the prognostic power of the pyroptosis risk score in patients with gastric cancer.** (A) Kaplan–Meier survival analysis showed the prognosis of PRS^low^ and PRS^high^ GC patients in GSE15459, GSE62254, GSE84437 cohorts. (B) Univariate and multivariate COX regression analyses were used to analyze the effect of PRS^low^ and PRS^high^ on overall survival in GSE15459, GSE62254, and GSE84437 cohorts.

**Figure S17.** Immunohistochemical scoring criteria for *BATF2*, *PTPRJ*, *RGS1*, and *VCAN*. Scale bar = 200 μm.

**Figure S18.** (A-E) Kaplan–Meier survival curves show the results of stratified analysis of gastric cancer patients with different clinicopathological characteristics in the five cohorts.

**Figure S19.** (A) Immunohistochemical scoring criteria for *BATF2*, *PTPRJ*, *RGS1*, and *VCAN* in gastric cancer (GC) tissue microarray. Scale bar = 200 μm. (B) Immunohistochemical scoring criteria for Caspase-1, Caspase-3, GSDMD, and GSDME in GC tissue microarray. Scale bar = 200 μm.

**Figure S20.** **The pyroptosis risk score demonstrated a significant association with pyroptosis in data from our center.** (A) Student’s *t*-test was used to compare the immunohistochemistry (IHC) scores of pyroptosis-related proteins (Caspase-1, Caspase-3, GSDMD, and GSDME) in pyroptosis risk score (PRS)^low^ and PRS^high^ groups. The upper and lower ends of the box indicate the interquartile range of values. The lines in the box indicate the median. (B) Scatter plots show Pearson’s correlation of PRS with IHC scores of pyroptosis-related proteins. (C) The integrated heatmap shows the mRNA expression levels of 24 pyroptosis-related proteins in the PRS^low^ and PRS^high^ groups in the FMUUN_RNA-Seq cohort. (D) Comprehensive gene set enrichment analysis revealed pathways enriched in PRS^low^ and PRS^high^ groups in the FMUUN_RNA-Seq cohort. (D) Comprehensive gene set enrichment analysis revealed pathways enriched in PRS^low^ and PRS^high^ groups in the GSE15459, GSE54129, GSE62254, and TCGA cohorts.

**Figure S21. The correlation between the** **pyroptosis risk score and immunophenotype was examined using data from our center.** (A) Unsupervised clustering using the single-sample gene set enrichment analysis algorithm based on immune cells in the FMUUN_RNA-Seq cohort was divided into three clusters. (B) Schematic diagram of quantitative analysis of immune markers obtained by immunohistochemical staining of gastric cancer tissues. The tumor core (CT) is shown in red, and the invasive margin (IM) is shown in green. The average density of five representative CT and IM regions was used as the density of this tumor. Scale = 200 µm. (C) Representative immunohistochemistry images showed CD8 staining of immune-inflamed, immune-excluded, and immune-desert types. Scale bar = 200 µm.

**Figure S22.** **The spatial distribution characteristics and functions of T cells and tumor-associated macrophages, as reflected by the pyroptosis risk score (PRS), were evaluated through immunohistochemical staining.** (A) Immunohistochemical staining of immune cells (CD4^+^, CD45^+^, CD3^+^, CD8^+^, CD45RO^+^, and FOXP3^+^). The left panel is the scene of the center of the tumor (CT), and the right panel is the scene of the invasive margin (IM). Scale bar = 200 µm.(B) Comparison of immune infiltration in PRS^low^ versus PRS^high^ in CT or IM. Student’s *t*-test. Data were analyzed using Student’s *t*-test. The upper and lower ends of the box indicate the interquartile range of values. The lines in the box indicate the median and the dots indicate outliers. (C) Correlation of pyroptosis risk score (PRS) with immune infiltration in CT and IM. Spearman test. (D) (A) Immunohistochemical staining for tumor-associated macrophages (CD68^+^ and CD206^+^). The upper panel shows the scene of the CT and the lower panel shows the IM. Scale bar = 200 µm. The lower panel compares the tumor-associated macrophage infiltration of PRS^low^ versus PRS^high^ in CT or IM. Box plots demonstrate tumor-associated macrophage infiltration of PRS^low^ versus PRS^high^ in CT or IM. Data were analyzed using Student’s *t*-test. The upper and lower ends of the box indicate the interquartile range of values. The lines in the box indicate the median and the dots indicate outliers. (E) Correlation of PRS with tumor-associated macrophage infiltration in CT and IM. Significance was tested using Spearman’s test.

**Figure S23.** **The expression levels of marker genes and key** **pyroptosis risk score genes in each cell cluster are depicted.** (A) Unsupervised clustering analysis of single-cell sequencing transcriptome profiles of tumor cells using marker genes defined 14 unique cell clusters, as well as the activated Gene Ontology- and KEGG-enriched pathways involved in the 14 cell clusters. (See Supplementary File 3 Single-cell sequencing differentially expressed genes and pathways for details.) (B) Characteristic gene expression patterns of the corresponding cell clusters on the t-SNE plot. (C-F) These plots demonstrate the expression of specific genes in the corresponding cell clusters. *BATF2* was mainly expressed in epithelial cells. *PTPRJ* was mainly expressed in epithelial cells, effector T cells, CD4+ T cells, and macrophages. *RGS1* was widely expressed in endocrine and parietal cells. *VCAN* was significantly expressed in fibroblasts, monocytes, and macrophages.

**Figure S24. The infiltration and spatial distribution of immune cells associated with pyroptosis risk score were revealed through single-cell sequencing and multiplex immunofluorescence staining.** (A) The percentage of B cells, DC, endocrine cells, endothelial cells, epithelial cells, plasmacytes, fibroblasts, macrophages, mast cells, monocytes, pericytes, and proliferative cells were compared between pyroptosis risk score (PRS)^low^ (n = 4) and PRS^high^ (n = 4). They consisted of four samples with the highest PRS (Sample01T, PRS = 74.321; Sample03T, PRS = 77.978; Sample06T, PRS = 88.530; Sample08T, PRS = 76.615) and four samples with the lowest PRS (Sample02T, PRS = -48.94; Sample04T, PRS = -49.564; Sample05T, PRS = -40.841; Sample07T, PRS = -42.742). Data are presented as the mean ± SD and were analyzed using Student’s *t*-test. (B) Box plots show the ratio (to total CD8^+^ cells) of Effector T cells (Teffs) in PRS^low^ and PRS^high^, as well as their distribution at different sites in the tumor nest and stroma. (Data were analyzed using Student’s *t*-test; The upper and lower ends of the box indicate the interquartile range of values. The lines in the box indicate the median and each dot signifies the corresponding value obtained from individual samples; PRS^low^: n = 13, PRS^high^: n = 13). (C) Representative images show the expression of tumor-associated macrophages in the invasive margin (IM) in both PRS^high^ and PRS^low^ groups on multiple immunofluorescence staining (CD68-green, CD206-red, iNOS-yellow, panCK-white, and DAPI-blue; n = 26; Scale bar = 100 μm). (D) Box plots show the densities of macrophages in PRS^low^ and PRS^high^ in the CT and IM. (Data were analyzed using Student’s *t*-test; The upper and lower ends of the box indicate the interquartile range of values. The lines in the box indicate the median and the dots indicate outliers; PRS^low^: n = 13, PRS^high^: n = 13).

**Figure S25.** **The pyroptosis risk score was utilized to assess the therapeutic efficacy in patients who received neoadjuvant chemotherapy only.** (A) Tumor regression grade (TRG) composition after neoadjuvant chemotherapy was compared between pyroptosis risk score (PRS)^low^ and PRS^high^ patients. (B) PRS was compared between patients who benefited from neoadjuvant chemotherapy and those who did not. Data were analyzed using Student’s *t*-test. The upper and lower ends of the boxes indicate the interquartile range of values. The lines in the boxes indicate the median. (C) Objective response after neoadjuvant chemotherapy was compared between PRS^low^ patients and PRS^high^ patients. (D) PRS was compared between patients who benefited from neoadjuvant chemotherapy and those who did not. Data were analyzed using Student’s *t*-test. The upper and lower ends of the box indicate the interquartile range of values. The lines in the box indicate the median. (E) Kaplan–Meier survival analysis showed recurrence-free survival in PRS^low^ patients compared with PRS^high^ patients. *p*-value survival analyses were calculated using the log-rank test. (F) The receiver operating characteristic curve was used to compare the accuracy of biomarkers (PRS, CPS, and inflammatory phenotype) in predicting response to neoadjuvant chemotherapy. (G) The predictive value of PRS for programmed cell death 1 (PD-1)-targeted therapy in patients with gastric cancer was validated in the PRJEB25780 cohort. The left panel shows the PRS comparison between patients who benefited from immunotherapy and those who did not. Data were analyzed using Student’s *t*-test. The upper and lower ends of the boxes indicate the interquartile range of values. The lines in the boxes indicate the median. The right panel shows the objective response after immunotherapy between PRS^low^ and PRS^high^ patients. (H) Comparison of benefits between the group receiving neoadjuvant immunotherapy and the group receiving neoadjuvant chemotherapy. *, *p* <0.05; **, *p* <0.01; ***, *p* <0.001. (I) Typical representative image of PD-L1 immunohistochemical staining. Scale bar= 200 um. ICI, immune checkpoint inhibitor; CT, chemotherapy.

**Figure S26. All patients receiving neoadjuvant therapy were stratified based on their pyroptosis risk score and the combined positive score (CPS).** (A-E) The tumor regression grade (TRG) composition and objective response after neoadjuvant immunotherapy or neoadjuvant chemotherapy alone were compared. ICI, immune checkpoint inhibitor; CT, chemotherapy.
