# Supplementary material for: Comprehensive multi-omics analysis of pyroptosis for optimizing neoadjuvant immunotherapy in patients with gastric cancer
Source: Theranostics. 2024 May 5;14(7):2915–33. doi: 10.7150/thno.93124 (PMC11103507; doi:10.7150/thno.93124)
Supplement: Supplementary file 1 — Supplementary figures and tables. [file thnov14p2915s1.zip › Supplementary figures and tables/Table S1.docx]

**Table S1. Clinicopathological Characteristics of the GC Patients in Validation-1 FJMUUH1 Cohort.** **(n=361)**

| **Variables** | **Total** | **PRS** | | | |
| --- | --- | --- | --- | --- | --- |
|  |  | **low** | **high** | ***χ*2** | ***P*** |
| **Gender** |  |  |  | 0.000 | 1.000 |
| Male | 270 | 135 | 135 |  |  |
| Female | 91 | 45 | 46 |  |  |
| **Age at surgery (years)** |  |  |  | 0.000 | 1.000 |
| ≥65 | 157 | 78 | 79 |  |  |
| <65 | 204 | 102 | 102 |  |  |
| **BMI** |  |  |  | 1.244 | 0.265 |
| <25 | 302 | 155 | 147 |  |  |
| ≥25 | 59 | 25 | 34 |  |  |
| **Chemotherapy** |  |  |  | 0.336 | 0.562 |
| No | 179 | 86 | 93 |  |  |
| Yes | 182 | 94 | 88 |  |  |
| **Depth of invasion** |  |  |  | 9.022 | **0.029** |
| T1 | 23 | 14 | 9 |  |  |
| T2 | 43 | 29 | 14 |  |  |
| T3 | 132 | 66 | 66 |  |  |
| T4 | 163 | 71 | 92 |  |  |
| **Lymph node metastasis** |  |  |  | 18.843 | **0.000** |
| N0 | 73 | 49 | 24 |  |  |
| N1 | 63 | 37 | 26 |  |  |
| N2 | 85 | 41 | 44 |  |  |
| N3 | 140 | 53 | 87 |  |  |
| **Distant metastasis** |  |  |  | 3.222 | 0.073 |
| M0 | 356 | 180 | 176 |  |  |
| M1 | 5 | 0 | 5 |  |  |
| **TNM stage** |  |  |  | 19.489 | **0.000** |
| I | 36 | 24 | 12 |  |  |
| II | 98 | 61 | 37 |  |  |
| III | 222 | 95 | 127 |  |  |
| IV | 5 | 0 | 5 |  |  |

*P* < 0.05 marked in bold font shows statistical significance.
