# Supplementary material for: Comprehensive multi-omics analysis of pyroptosis for optimizing neoadjuvant immunotherapy in patients with gastric cancer
Source: Theranostics. 2024 May 5;14(7):2915–33. doi: 10.7150/thno.93124 (PMC11103507; doi:10.7150/thno.93124)
Supplement: Supplementary file 1 — Supplementary figures and tables. [file thnov14p2915s1.zip › Supplementary figures and tables/Table S10.docx]

**Table S10. Cox regression analysis of prognostic factors for prognosis.**

| **Variables** | **Validation-5 South China Cohort (n=166)** | | | | | | |
| --- | --- | --- | --- | --- | --- | --- | --- |
|  | **Univariate analysis** | | | | **Multivariate analysis** | | |
|  | **HR** | **95% CI** | | ***P*** | **HR** | **95% CI** | ***P*** |
| PRS (high vs <low) | 3.212 | | 1.923-5.366 | **<0.001** | 2.806 | 1.661-4.74 | **<0.001** |
| Age (≥65 vs <65) | 1.291 | | 0.799-2.085 | 0.296 |  |  |  |
| Gender (male vs female) | 0.876 | | 0.547-1.4 | 0.579 |  |  |  |
| BMI (≥25 vs <25) | 0.837 | | 0.426-1.646 | 0.606 |  |  |  |
| pT Stage (T3\T4 vs T1\T2) | 3.602 | | 1.789-7.251 | **<0.001** | 2.174 | 0.895-5.282 | 0.086 |
| pN Stage (N2\N3 vs N0\N1) | 2.278 | | 1.304-3.978 | **0.004** | 1.293 | 0.634-2.637 | 0.48 |
| pTNM Stage (III\IV vs I\II) | 3.273 | | 1.915-5.592 | **<0.001** | 1.541 | 0.674-3.523 | 0.306 |
| Tumor Size (≥5mm vs <5mm) | 1.668 | | 0.995-2.796 | 0.052 |  |  |  |
| CA199 (elevated vs normal) | 5.537 | | 2.121-14.46 | **<0.001** |  |  |  |
| CEA (elevated vs normal) | 1.998 | | 0.694-5.752 | 0.199 |  |  |  |

*P* < 0.05 marked in bold font shows statistical significance.
