# Supplementary material for: Comprehensive multi-omics analysis of pyroptosis for optimizing neoadjuvant immunotherapy in patients with gastric cancer
Source: Theranostics. 2024 May 5;14(7):2915–33. doi: 10.7150/thno.93124 (PMC11103507; doi:10.7150/thno.93124)
Supplement: Supplementary file 1 — Supplementary figures and tables. [file thnov14p2915s1.zip › Supplementary figures and tables/Table S11.docx]

**Table S11. Clinicopathological Characteristics of Neoadjuvant ICI Therapy Combined with Chemotherapy versus Neoadjuvant Chemotherapy Only in the Patients.**

| **Variables** | **Group** | | | |
| --- | --- | --- | --- | --- |
|  | **Neoadjuvant ICI with CT** | **Neoadjuvant CT Only** | ***χ*2** | ***P*** |
| **Response** |  |  | 1.239 | 0.265 |
| CR/PR | 24 | 18 |  |  |
| SD/PD | 24 | 31 |  |  |
| **TRG** |  |  | 1.640 | 0.200 |
| 1a/1b | 16 | 10 |  |  |
| 2/3 | 30 | 38 |  |  |
| **ypT Stage** |  |  | 1.429 | 0.232 |
| T0/T1 | 14 | 9 |  |  |
| T2/T3 | 31 | 40 |  |  |
| **ypN Stage** |  |  | 0.221 | 0.638 |
| N0 | 21 | 19 |  |  |
| N1 | 25 | 30 |  |  |
| **pTNM stage** |  |  | 2.660 | 0.103 |
| pCR/I | 17 | 10 |  |  |
| II/III | 28 | 39 |  |  |

*P* < 0.05 marked in bold font shows statistical significance.
