# Supplementary material for: Comprehensive multi-omics analysis of pyroptosis for optimizing neoadjuvant immunotherapy in patients with gastric cancer
Source: Theranostics. 2024 May 5;14(7):2915–33. doi: 10.7150/thno.93124 (PMC11103507; doi:10.7150/thno.93124)
Supplement: Supplementary file 1 — Supplementary figures and tables. [file thnov14p2915s1.zip › Supplementary figures and tables/Table S12.docx]

**Table S12. Clinicopathological Characteristics of the Neoadjuvant Chemotherapy in Patients.**

| **Variables** | **Total** | **PRS** | | | |
| --- | --- | --- | --- | --- | --- |
|  |  | **low** | **high** | ***χ*2** | ***P*** |
| **Response** |  |  |  | 0.996 | 0.318 |
| CR/PR | 18 | 11 | 7 |  |  |
| SD/PD | 31 | 13 | 18 |  |  |
| **TRG** |  |  |  | 0.126 | 0.722 |
| 1a/1b | 10 | 6 | 4 |  |  |
| 2/3 | 38 | 18 | 20 |  |  |
| **ypT Stage** |  |  |  | 0.649 | 0.420 |
| T0/T1 | 9 | 6 | 3 |  |  |
| T2/T3 | 40 | 18 | 22 |  |  |
| **ypN Stage** |  |  |  | 0.490 | 0.484 |
| N0 | 19 | 11 | 8 |  |  |
| N1 | 30 | 13 | 17 |  |  |
| **pTNM stage** |  |  |  | 0.669 | 0.182 |
| pCR/I | 10 | 6 | 4 |  |  |
| II/III | 39 | 18 | 21 |  |  |

*P* < 0.05 marked in bold font shows statistical significance.
