# Supplementary material for: Comprehensive multi-omics analysis of pyroptosis for optimizing neoadjuvant immunotherapy in patients with gastric cancer
Source: Theranostics. 2024 May 5;14(7):2915–33. doi: 10.7150/thno.93124 (PMC11103507; doi:10.7150/thno.93124)
Supplement: Supplementary file 1 — Supplementary figures and tables. [file thnov14p2915s1.zip › Supplementary figures and tables/Table S13.docx]

| **Table S13. Retrieval of pyroptosis-related genes** | |
| --- | --- |
| **Genes** | **Iiterature source** |
| AIM2 | [1] Lee S, Karki R, Wang Y, et al. AIM2 forms a complex with Pyrin and ZBP1 to drive PANoptosis and host defense[J]. Nature, 2021, 597(7876): 415–419. [2] Sharma B R, Karki R, Kanneganti T-D. Role of AIM2 inflammasome in inflammatory diseases, cancer and infection[J]. European journal of immunology, 2019, 49(11): 1998–2011. [3] Lugrin J, Martinon F. The AIM2 inflammasome: Sensor of pathogens and cellular perturbations[J]. Immunological Reviews, 2018, 281(1): 99–114. |
| CASP1 | [4] Wree A, Eguchi A, McGeough M D, et al. NLRP3 inflammasome activation results in hepatocyte pyroptosis, liver inflammation, and fibrosis in mice[J]. Hepatology (Baltimore, Md.), 2014, 59(3): 898–910. [5] Guo H, Callaway J B, Ting J P-Y. Inflammasomes: mechanism of action, role in disease, and therapeutics[J]. Nature Medicine, 2015, 21(7): 677–687. [6] Tsuchiya K, Nakajima S, Hosojima S, et al. Caspase-1 initiates apoptosis in the absence of gasdermin D[J]. Nature Communications, 2019, 10(1): 2091. |
| CASP3 | [7] Wang Y, Gao W, Shi X, et al. Chemotherapy drugs induce pyroptosis through caspase-3 cleavage of a gasdermin[J]. Nature, 2017, 547(7661): 99–103. [8] Li Y, Yuan Y, Huang Z-X, et al. GSDME-mediated pyroptosis promotes inflammation and fibrosis in obstructive nephropathy[J]. Cell Death and Differentiation, 2021, 28(8): 2333–2350. [9] Jiang M, Qi L, Li L, et al. The caspase-3/GSDME signal pathway as a switch between apoptosis and pyroptosis in cancer[J]. Cell Death Discovery, 2020, 6: 112. |
| CASP4 | [10] Liu X, Zhang Z, Ruan J, et al. Inflammasome-activated gasdermin D causes pyroptosis by forming membrane pores[J]. Nature, 2016, 535(7610): 153–158. [11] Shi J, Zhao Y, Wang K, et al. Cleavage of GSDMD by inflammatory caspases determines pyroptotic cell death[J]. Nature, 2015, 526(7575): 660–665. |
| CASP5 | [12] Shi J, Zhao Y, Wang Y, et al. Inflammatory caspases are innate immune receptors for intracellular LPS[J]. Nature, 2014, 514(7521): 187–192. [13] Jorgensen I, Miao E A. Pyroptotic cell death defends against intracellular pathogens[J]. Immunological reviews, 2015, 265(1): 130–142. |
| CASP6 | [14] Zheng M, Karki R, Kancharana B, et al. Caspase-6 promotes activation of the caspase-11-NLRP3 inflammasome during gram-negative bacterial infections[J]. The Journal of Biological Chemistry, 2021, 297(6): 101379. [15] Zheng M, Karki R, Vogel P, et al. Caspase-6 Is a Key Regulator of Innate Immunity, Inflammasome Activation, and Host Defense[J]. Cell, 2020, 181(3): 674-687.e13. |
| CASP8 | [16] Fritsch M, Günther S D, Schwarzer R, et al. Caspase-8 is the molecular switch for apoptosis, necroptosis and pyroptosis[J]. Nature, 2019, 575(7784): 683–687. [17] Man S M, Kanneganti T-D. Converging roles of caspases in inflammasome activation, cell death and innate immunity[J]. Nature Reviews. Immunology, 2016, 16(1): 7–21. |
| DHX9 | [18] Ngo C, Man S M. NLRP9b: a novel RNA-sensing inflammasome complex[J]. Cell Research, 2017, 27(11): 1302–1303. [19] Zhu S, Ding S, Wang P, et al. Nlrp9b inflammasome restricts rotavirus infection in intestinal epithelial cells[J]. Nature, 2017, 546(7660): 667–670. |
| DPP8 | [20] Okondo M C, Johnson D C, Sridharan R, et al. DPP8 and DPP9 inhibition induces pro-caspase-1-dependent monocyte and macrophage pyroptosis[J]. Nature Chemical Biology, 2017, 13(1): 46–53. [21] Johnson D C, Taabazuing C Y, Okondo M C, et al. DPP8/9 inhibitor-induced pyroptosis for treatment of acute myeloid leukemia[J]. Nature medicine, 2018, 24(8): 1151–1156. |
| GSDMB | [22] Deng W, Bai Y, Deng F, et al. Streptococcal pyrogenic exotoxin B cleaves GSDMA and triggers pyroptosis[J]. Nature, 2022, 602(7897): 496–502.  [23] Hou J, Hsu J-M, Hung M-C. Molecular mechanisms and functions of pyroptosis in inflammation and antitumor immunity[J]. Molecular Cell, 2021, 81(22): 4579–4590.  [24] Zhou Z, He H, Wang K, et al. Granzyme A from cytotoxic lymphocytes cleaves GSDMB to trigger pyroptosis in target cells[J]. Science (New York, N.Y.), 2020, 368(6494): eaaz7548. |
| GSDMD | [25] Ding J, Wang K, Liu W, et al. Pore-forming activity and structural autoinhibition of the gasdermin family[J]. Nature, 2016, 535(7610): 111–116. [26] Chen K W, Demarco B, Broz P. Beyond inflammasomes: emerging function of gasdermins during apoptosis and NETosis[J]. The EMBO journal, 2020, 39(2): e103397. [27] Chen K W, Demarco B, Heilig R, et al. Extrinsic and intrinsic apoptosis activate pannexin-1 to drive NLRP3 inflammasome assembly[J]. The EMBO journal, 2019, 38(10): e101638. |
| GSDME | [28] Zhang Z, Zhang Y, Xia S, et al. Gasdermin E suppresses tumour growth by activating anti-tumour immunity[J]. Nature, 2020, 579(7799): 415–420. [29] Hu Y, Wen Q, Cai Y, et al. Alantolactone induces concurrent apoptosis and GSDME-dependent pyroptosis of anaplastic thyroid cancer through ROS mitochondria-dependent caspase pathway[J]. Phytomedicine: International Journal of Phytotherapy and Phytopharmacology, 2023, 108: 154528. |
| GZMA | [30] Zhu P, Martinvalet D, Chowdhury D, et al. The cytotoxic T lymphocyte protease granzyme A cleaves and inactivates poly(adenosine 5′-diphosphate-ribose) polymerase-1[J]. Blood, 2009, 114(6): 1205–1216. [31] Zhou Z, He H, Wang K, et al. Granzyme A from cytotoxic lymphocytes cleaves GSDMB to trigger pyroptosis in target cells[J]. Science, 2020, 368(6494): eaaz7548. [32] Lieberman J. Granzyme A activates another way to die: Granzyme A-mediated cell death[J]. Immunological Reviews, 2010, 235(1): 93–104. |
| GZMB | [33] Turner C T, Hiroyasu S, Granville D J. Granzyme B as a therapeutic target for wound healing[J]. Expert Opinion on Therapeutic Targets, 2019, 23(9): 745–754. [34] Velotti F, Barchetta I, Cimini F A, et al. Granzyme B in Inflammatory Diseases: Apoptosis, Inflammation, Extracellular Matrix Remodeling, Epithelial-to-Mesenchymal Transition and Fibrosis[J]. Frontiers in Immunology, 2020, 11: 587581. |
| HMGB1 | [35] Deng M, Tang Y, Li W, et al. The Endotoxin Delivery Protein HMGB1 Mediates Caspase-11-Dependent Lethality in Sepsis[J]. Immunity, 2018, 49(4): 740-753.e7. [36] Zhu D, Zou H, Liu J, et al. Inhibition of HMGB1 Ameliorates the Maternal-Fetal Interface Destruction in Unexplained Recurrent Spontaneous Abortion by Suppressing Pyroptosis Activation[J]. Frontiers in Immunology, 2021, 12: 782792. [37] Volchuk A, Ye A, Chi L, et al. Indirect regulation of HMGB1 release by gasdermin D[J]. Nature Communications, 2020, 11(1): 4561. [38] Tan G, Huang C, Chen J, et al. HMGB1 released from GSDME-mediated pyroptotic epithelial cells participates in the tumorigenesis of colitis-associated colorectal cancer through the ERK1/2 pathway[J]. Journal of Hematology & Oncology, 2020, 13(1): 149. [39] Geng Y, Ma Q, Liu Y-N, et al. Heatstroke induces liver injury via IL-1β and HMGB1-induced pyroptosis[J]. Journal of Hepatology, 2015, 63(3): 622–633. |
| IL-1β | [40] Muendlein H I, Jetton D, Connolly W M, et al. cFLIP L protects macrophages from LPS-induced pyroptosis via inhibition of complex II formation[J]. Science, 2020, 367(6484): 1379–1384.[41] He W, Wan H, Hu L, et al. Gasdermin D is an executor of pyroptosis and required for interleukin-1β secretion[J]. Cell Research, 2015, 25(12): 1285–1298.[42] Geng Y, Ma Q, Liu Y-N, et al. Heatstroke induces liver injury via IL-1β and HMGB1-induced pyroptosis[J]. Journal of Hepatology, 2015, 63(3): 622–633.[43] Gaidt M M, Ebert T S, Chauhan D, et al. Human Monocytes Engage an Alternative Inflammasome Pathway[J]. Immunity, 2016, 44(4): 833–846.[44] Karmakar M, Minns M, Greenberg E N, et al. N-GSDMD trafficking to neutrophil organelles facilitates IL-1β release independently of plasma membrane pores and pyroptosis[J]. Nature Communications, 2020, 11(1): 2212. |
| IL-18 | [45] Wittmann M, Macdonald A, Renne J. IL-18 and skin inflammation[J]. Autoimmunity Reviews, 2009, 9(1): 45–48. [46] Hirata J, Kotani J, Aoyama M, et al. A ROLE FOR IL-18 IN HUMAN NEUTROPHIL APOPTOSIS[J]. Shock, 2008, 30(6): 628–633. |
| NAIP | [47] Yang J, Zhao Y, Shi J, et al. Human NAIP and mouse NAIP1 recognize bacterial type III secretion needle protein for inflammasome activation[J]. Proceedings of the National Academy of Sciences, 2013, 110(35): 14408–14413. |
| NLRP1 | [48] Mi L, Min X, Chai Y, et al. NLRP1 Inflammasomes: A Potential Target for the Treatment of Several Types of Brain Injury[J]. Frontiers in Immunology, 2022, 13: 863774. [49] Kovarova M, Hesker P R, Jania L, et al. NLRP1-Dependent Pyroptosis Leads to Acute Lung Injury and Morbidity in Mice[J]. The Journal of Immunology, 2012, 189(4): 2006–2016. [50] Chavarría-Smith J, Vance R E. The NLRP1 inflammasomes[J]. Immunological Reviews, 2015, 265(1): 22–34. |
| NLRP3 | [51] Chen H, Deng Y, Gan X, et al. NLRP12 collaborates with NLRP3 and NLRC4 to promote pyroptosis inducing ganglion cell death of acute glaucoma[J]. Molecular Neurodegeneration, 2020, 15(1): 26. [52] Faria S S, Costantini S, De Lima V C C, et al. NLRP3 inflammasome-mediated cytokine production and pyroptosis cell death in breast cancer[J]. Journal of Biomedical Science, 2021, 28(1): 26. [53] Huang Y, Xu W, Zhou R. NLRP3 inflammasome activation and cell death[J]. Cellular & Molecular Immunology, 2021, 18(9): 2114–2127. [54] Gao L, Dong X, Gong W, et al. Acinar cell NLRP3 inflammasome and gasdermin D (GSDMD) activation mediates pyroptosis and systemic inflammation in acute pancreatitis[J]. British Journal of Pharmacology, 2021, 178(17): 3533–3552. |
| PYCARD | [55] Miao H, Wang L, Zhan H, et al. A long noncoding RNA distributed in both nucleus and cytoplasm operates in the PYCARD-regulated apoptosis by coordinating the epigenetic and translational regulation[J]. K.V. Morris. PLOS Genetics, 2019, 15(5): e1008144. |
| STAT3 | [56] Feng Y, Li W, Wang Z, et al. The p-STAT3/ANXA2 axis promotes caspase-1-mediated hepatocyte pyroptosis in non-alcoholic steatohepatitis[J]. Journal of Translational Medicine, 2022, 20(1): 497. [57] Yang Z, Wang Y, Liu Q, et al. microRNA cluster MC‐let‐7a‐1~let‐7d promotes autophagy and apoptosis of glioma cells by down‐regulating STAT3[J]. CNS Neuroscience & Therapeutics, 2020, 26(3): 319–331. [58] Liu K, Ren T, Huang Y, et al. Apatinib promotes autophagy and apoptosis through VEGFR2/STAT3/BCL-2 signaling in osteosarcoma[J]. Cell Death & Disease, 2017, 8(8): e3015–e3015. |
| TREM2 | [59] Wang Y, Cao C, Zhu Y, et al. TREM2/β-catenin attenuates NLRP3 inflammasome-mediated macrophage pyroptosis to promote bacterial clearance of pyogenic bacteria[R]. In Review, 2022. |
| ZBP1 | [60] Lee S, Karki R, Wang Y, et al. AIM2 forms a complex with pyrin and ZBP1 to drive PANoptosis and host defence[J]. Nature, 2021, 597(7876): 415–419.[61] Zhang T, Yin C, Boyd D F, et al. Influenza Virus Z-RNAs Induce ZBP1-Mediated Necroptosis[J]. Cell, 2020, 180(6): 1115-1129.e13.[62] Jiao H, Wachsmuth L, Kumari S, et al. Z-nucleic-acid sensing triggers ZBP1-dependent necroptosis and inflammation[J]. Nature, 2020, 580(7803): 391–395. |
