# Supplementary material for: Comprehensive multi-omics analysis of pyroptosis for optimizing neoadjuvant immunotherapy in patients with gastric cancer
Source: Theranostics. 2024 May 5;14(7):2915–33. doi: 10.7150/thno.93124 (PMC11103507; doi:10.7150/thno.93124)
Supplement: Supplementary file 1 — Supplementary figures and tables. [file thnov14p2915s1.zip › Supplementary figures and tables/Table S14.docx]

**Table S14. Computational tools used in tumor immunogenomics with high-throughput next-generation sequencing data.**

| Tool | Characteristics | URL | Year |
| --- | --- | --- | --- |
| CIBERSORT | Based on linear support vector regression, deconvolution from microarray data, and known gene expression profiling set. | https://cibersort.stanford.edu/ | 2015 |
| CIBERSORTx | Expanding data source to single-cell RNA-seq. | https://cibersortx.stanford.edu/ | 2019 |
| EPIC | Based on constrained least square, incorporates a non-negative condition into deconvolution. | https://gfellerlab.shinyapps.io/EPIC_1-1/ | 2017 |
| ESTIMATE | Generating a stromal score and immune score to reflect the tumor purity based on ssGSEA | https://sourceforge.net/projects/estimateproject/ | 2013 |
| MCP-counter | The score is the geometric mean of the expression level of cell-specific genes, implying the absolute abundance of immune cell types among samples | http://github.com/ebecht/MCPcounter | 2016 |
| quanTIseq | Quantification of ten different immune cell types and other uncharacterized cells based on RNA-seq data | http://icbi.at/quantiseq | 2019 |
| TIMER | Immune cells are estimated via transcriptomic data and the correlations among the immunological, genomic, and clinical features were established. | https://cistrome.shinyapps.io/timer/ | 2016 |
| xCell | Spillover compensation is used to separate cell types with high correlation | http://xcell.ucsf.edu/ | 2017 |
