# Supplementary material for: Comprehensive multi-omics analysis of pyroptosis for optimizing neoadjuvant immunotherapy in patients with gastric cancer
Source: Theranostics. 2024 May 5;14(7):2915–33. doi: 10.7150/thno.93124 (PMC11103507; doi:10.7150/thno.93124)
Supplement: Supplementary file 1 — Supplementary figures and tables. [file thnov14p2915s1.zip › Supplementary figures and tables/Table S2.docx]

**Table S2. Clinicopathological Characteristics of the GC Patients in Validation-1 FJMUUH2 Cohort.** **(n=253)**

| **Variables** | **Total** | **PRS** | | | |
| --- | --- | --- | --- | --- | --- |
|  |  | **low** | **high** | ***χ*2** | ***P*** |
| **Gender** |  |  |  | 0.297 | 0.586 |
| Male | 190 | 97 | 93 |  |  |
| Female | 63 | 29 | 34 |  |  |
| **Age at surgery (years)** |  |  |  | 0.043 | 0.836 |
| ≥65 | 99 | 48 | 51 |  |  |
| <65 | 154 | 78 | 76 |  |  |
| **Chemotherapy** |  |  |  | 0.056 | 0.812 |
| No | 73 | 35 | 38 |  |  |
| Yes | 180 | 91 | 89 |  |  |
| **Depth of invasion** |  |  |  | 6.453 | 0.092 |
| T1 | 31 | 14 | 17 |  |  |
| T2 | 21 | 6 | 15 |  |  |
| T3 | 96 | 46 | 50 |  |  |
| T4 | 105 | 60 | 45 |  |  |
| **Lymph node metastasis** |  |  |  | 17.27 | **0.001** |
| N0 | 60 | 24 | 36 |  |  |
| N1 | 33 | 8 | 25 |  |  |
| N2 | 55 | 29 | 26 |  |  |
| N3 | 105 | 65 | 40 |  |  |
| **TNM stage** |  |  |  | 14.912 | **0.001** |
| I | 38 | 17 | 21 |  |  |
| II | 51 | 14 | 37 |  |  |
| III | 164 | 95 | 69 |  |  |
| **MSI status** |  |  |  | 0.063 | 0.801 |
| MSI High | 20 | 11 | 9 |  |  |
| MSI Low/MSS | 233 | 115 | 118 |  |  |
| **EBV status** |  |  |  | 0.000 | 1.000 |
| Negative | 232 | 116 | 116 |  |  |
| Positive | 21 | 10 | 11 |  |  |

*P* < 0.05 marked in bold font shows statistical significance.
