# Supplementary material for: Comprehensive multi-omics analysis of pyroptosis for optimizing neoadjuvant immunotherapy in patients with gastric cancer
Source: Theranostics. 2024 May 5;14(7):2915–33. doi: 10.7150/thno.93124 (PMC11103507; doi:10.7150/thno.93124)
Supplement: Supplementary file 1 — Supplementary figures and tables. [file thnov14p2915s1.zip › Supplementary figures and tables/Table S3.docx]

**Table S3. Clinicopathological Characteristics of the GC Patients in North China Cohort Cohort.** **(n=98)**

| **Variables** | **Total** | **PRS** | | | |
| --- | --- | --- | --- | --- | --- |
|  |  | **low** | **high** | ***χ*2** | ***P*** |
| **Gender** |  |  |  | 0.45 | 0.502 |
| Male | 70 | 33 | 37 |  |  |
| Female | 28 | 16 | 12 |  |  |
| **Age at surgery (years)** |  |  |  | 0.042 | 0.837 |
| ≥65 | 40 | 21 | 19 |  |  |
| <65 | 58 | 28 | 30 |  |  |
| **BMI** |  |  |  | 0.000 | 1.000 |
| <25 | 63 | 32 | 31 |  |  |
| ≥25 | 35 | 17 | 18 |  |  |
| **Chemotherapy** |  |  |  | 0.215 | 0.643 |
| No | 25 | 11 | 14 |  |  |
| Yes | 73 | 38 | 35 |  |  |
| **Depth of invasion** |  |  |  | 3.034 | 0.386 |
| T1 | 12 | 7 | 5 |  |  |
| T2 | 13 | 9 | 4 |  |  |
| T3 | 10 | 5 | 5 |  |  |
| T4 | 63 | 28 | 35 |  |  |
| **Lymph node metastasis** |  |  |  | 0.611 | 0.894 |
| N0 | 43 | 22 | 21 |  |  |
| N1 | 19 | 8 | 11 |  |  |
| N2 | 21 | 11 | 10 |  |  |
| N3 | 15 | 8 | 7 |  |  |
| **TNM stage** |  |  |  | 1.312 | 0.519 |
| I | 20 | 12 | 8 |  |  |
| II | 25 | 13 | 12 |  |  |
| III | 53 | 24 | 29 |  |  |

*P* < 0.05 marked in bold font shows statistical significance.
