# Supplementary material for: Comprehensive multi-omics analysis of pyroptosis for optimizing neoadjuvant immunotherapy in patients with gastric cancer
Source: Theranostics. 2024 May 5;14(7):2915–33. doi: 10.7150/thno.93124 (PMC11103507; doi:10.7150/thno.93124)
Supplement: Supplementary file 1 — Supplementary figures and tables. [file thnov14p2915s1.zip › Supplementary figures and tables/Table S4.docx]

**Table S4. Clinicopathological Characteristics of the GC Patients in Central China Cohort. (n=100)**

| **Variables** | **Total** | **PRS** | | | |
| --- | --- | --- | --- | --- | --- |
|  |  | **low** | **high** | ***χ*2** | ***P*** |
| **Gender** |  |  |  | 0.000 | 1.000 |
| Male | 77 | 38 | 39 |  |  |
| Female | 23 | 12 | 11 |  |  |
| **Age at surgery (years)** |  |  |  | 1.445 | 0.229 |
| ≥65 | 53 | 30 | 23 |  |  |
| <65 | 47 | 20 | 27 |  |  |
| **BMI** |  |  |  | 0.058 | 0.809 |
| <25 | 78 | 38 | 40 |  |  |
| ≥25 | 22 | 12 | 10 |  |  |
| **Chemotherapy** |  |  |  | 0.055 | 0.815 |
| No | 24 | 11 | 13 |  |  |
| Yes | 76 | 39 | 37 |  |  |
| **Depth of invasion** |  |  |  | 4.592 | 0.204 |
| T1 | 6 | 2 | 4 |  |  |
| T2 | 11 | 8 | 3 |  |  |
| T3 | 24 | 9 | 15 |  |  |
| T4 | 59 | 31 | 28 |  |  |
| **Lymph node metastasis** |  |  |  | 3.619 | 0.306 |
| N0 | 38 | 23 | 15 |  |  |
| N1 | 26 | 12 | 14 |  |  |
| N2 | 31 | 12 | 19 |  |  |
| N3 | 5 | 3 | 2 |  |  |
| **TNM stage** |  |  |  | 6.946 | 0.074 |
| I | 10 | 4 | 6 |  |  |
| II | 20 | 15 | 5 |  |  |
| III | 66 | 30 | 36 |  |  |
| IV | 4 | 1 | 3 |  |  |

*P* < 0.05 marked in bold font shows statistical significance.
