# Supplementary material for: Comprehensive multi-omics analysis of pyroptosis for optimizing neoadjuvant immunotherapy in patients with gastric cancer
Source: Theranostics. 2024 May 5;14(7):2915–33. doi: 10.7150/thno.93124 (PMC11103507; doi:10.7150/thno.93124)
Supplement: Supplementary file 1 — Supplementary figures and tables. [file thnov14p2915s1.zip › Supplementary figures and tables/Table S5.docx]

**Table S5. Clinicopathological Characteristics of the GC Patients in** **South China Cohort. (n=166).**

| **Variables** | **Total** | **PRS** | | | |
| --- | --- | --- | --- | --- | --- |
|  |  | **low** | **high** | ***χ*2** | ***P*** |
| **Gender** |  |  |  | 0.901 | 0.343 |
| Male | 99 | 46 | 53 |  |  |
| Female | 67 | 37 | 30 |  |  |
| **Age at surgery (years)** |  |  |  | 6.732 | **0.009** |
| ≥65 | 59 | 38 | 21 |  |  |
| <65 | 107 | 45 | 62 |  |  |
| **Chemotherapy** |  |  |  | 0.000 | 1.000 |
| No | 51 | 26 | 25 |  |  |
| Yes | 115 | 57 | 58 |  |  |
| **Depth of invasion** |  |  |  | 5.614 | 0.132 |
| T1 | 19 | 12 | 7 |  |  |
| T2 | 31 | 18 | 13 |  |  |
| T3 | 35 | 20 | 15 |  |  |
| T4 | 81 | 33 | 48 |  |  |
| **Lymph node metastasis** |  |  |  | 7.137 | 0.068 |
| N0 | 59 | 32 | 27 |  |  |
| N1 | 32 | 18 | 14 |  |  |
| N2 | 30 | 18 | 12 |  |  |
| N3 | 45 | 15 | 30 |  |  |
| **Distant metastasis** |  |  |  | 0.000 | 1.000 |
| M0 | 165 | 83 | 82 |  |  |
| M1 | 1 | 0 | 1 |  |  |
| **TNM stage** |  |  |  | 8.203 | **0.042** |
| I | 34 | 19 | 15 |  |  |
| II | 45 | 29 | 16 |  |  |
| III | 86 | 35 | 51 |  |  |
| IV | 1 | 0 | 1 |  |  |

*P* < 0.05 marked in bold font shows statistical significance.
