# Supplementary material for: Comprehensive multi-omics analysis of pyroptosis for optimizing neoadjuvant immunotherapy in patients with gastric cancer
Source: Theranostics. 2024 May 5;14(7):2915–33. doi: 10.7150/thno.93124 (PMC11103507; doi:10.7150/thno.93124)
Supplement: Supplementary file 1 — Supplementary figures and tables. [file thnov14p2915s1.zip › Supplementary figures and tables/Table S6.docx]

**Table S6. Cox regression analysis of prognostic factors for prognosis.**

| **Variables** |  |  | **Validation-1 FJMUUH1 (n=361)** | | | | | |
| --- | --- | --- | --- | --- | --- | --- | --- | --- |
|  |  |  | **Univariate analysis** | | | **Multivariate analysis** | | |
|  |  |  | **HR** | **95%CI** | ***P*** | **HR** | **95%CI** | ***P*** |
| PRS (high vs <low) | | | 4.2 2.966-5.948 **<0.001** | | | 3.577 2.519-5.079 **<0.001** | | |
| Age (≥65 vs <65) | | | 1.298 0.959-1.757 0.091 | | |  | | |
| Gender (male vs female) | | | 1.277 0.855-1.762 0.267 | | |  | | |
| BMI (≥25 vs <25) | | | 1.143 0.77-1.699 0.507 | | |  | | |
| pT Stage (T3\T4 vs T1\T2) | | | 3.702 2.099-6.526 **<0.001** | | | 2.043 1.051-3.971 **0.035** | | |
| pN Stage (N2\N3vs N0\N1) | | | 3.577 2.499-5.225  **<0.001** | | | 2.144 1.117-4.116 **0.022** | | |
| pTNM Stage (III\IV vs I\II) | | | 3.874 2.617-5.733 **<0.001** | | | 1.331 0.635-2.792 0.449 | | |

*P* < 0.05 marked in bold font shows statistical significance.
