# Supplementary material for: Comprehensive multi-omics analysis of pyroptosis for optimizing neoadjuvant immunotherapy in patients with gastric cancer
Source: Theranostics. 2024 May 5;14(7):2915–33. doi: 10.7150/thno.93124 (PMC11103507; doi:10.7150/thno.93124)
Supplement: Supplementary file 1 — Supplementary figures and tables. [file thnov14p2915s1.zip › Supplementary figures and tables/Table S7.docx]

**Table S7. Cox regression analysis of prognostic factors for prognosis.**

| **Variables** | **Validation-2 FJMUUH1 (n=253)** | | | | | | | |
| --- | --- | --- | --- | --- | --- | --- | --- | --- |
|  | **Univariate analysis** | | | | **Multivariate analysis** | | | |
|  | **HR** | **95% CI** | ***P*** | | **HR** | **95% CI** | ***P*** | |
| PRS (high vs <low) | 3.270 | 2.239-4.778 | | **<0.001** | 2.750 | 1.869-4.046 | | **<0.001** |
| Age (≥65 vs <65) | 1.19 | 0.839-1.689 | | 0.33 |  |  | |  |
| Gender (male vs female) | 0.897 | 0.604-1.333 | | 0.592 |  |  | |  |
| BMI (≥25 vs <25) | 0.785 | 0.455-1.354 | | 0.385 |  |  | |  |
| pT Stage (T3\T4 vs T1\T2) | 5.524 | 2.697-11.313 | | **<0.001** | 1.483 | 1.060-2.075 | | **0.022** |
| pN Stage (N2\N3 vs N0\N1) | 3.578 | 2.291-5.586 | | **<0.001** | 1.394 | 1.026-1.894 | | **0.034** |
| pTNM Stage (III\IV vs I\II) | 3.715 | 2.343-5.891 | | **<0.001** | 1.018 | 0.506-2.046 | | 0.961 |
| Tumor Size (≥5mm vs <5mm) | 2.629 | 1.767-3.912 | | **<0.001** |  |  | |  |
| CA199 (elevated vs normal) | 1.447 | 0.931-2.25 | | 0.101 |  |  | |  |
| CEA (elevated vs normal) | 1.11 | 0.595-2.072 | | 0.743 |  |  | |  |

*P* < 0.05 marked in bold font shows statistical significance.
