# Supplementary material for: Comprehensive multi-omics analysis of pyroptosis for optimizing neoadjuvant immunotherapy in patients with gastric cancer
Source: Theranostics. 2024 May 5;14(7):2915–33. doi: 10.7150/thno.93124 (PMC11103507; doi:10.7150/thno.93124)
Supplement: Supplementary file 1 — Supplementary figures and tables. [file thnov14p2915s1.zip › Supplementary figures and tables/Table S8.docx]

**Table S8. Cox regression analysis of prognostic factors for prognosis.**

| **Variables** | **Validation-3 North China Cohort (n=98)** | | | | | | | | | |
| --- | --- | --- | --- | --- | --- | --- | --- | --- | --- | --- |
|  | **Univariate analysis** | | | | | **Multivariate analysis** | | | | |
|  | **HR** | **95%CI** | ***P*** | | **HR** | | | **95%CI** | ***P*** | |
| PRS (high vs <low) | 3.054 | 1.283-7.269 | | **0.012** | | | 2.692 | 1.121-6.464 | | **0.027** |
| Age (≥65 vs <65) | 1.206 | 0.554-2.627 | | 0.637 | | |  |  | |  |
| Gender (male vs female) | 1.479 | 0.594-3.683 | | 0.401 | | |  |  | |  |
| BMI (≥25 vs <25) | 0.61 | 0.256-1.45 | | 0.263 | | |  |  | |  |
| pT Stage (T3\T4 vs T1\T2) | 4.949 | 1.169-20.956 | | **0.03** | | | 1.913 | 0.347-10.565 | | 0.457 |
| pN Stage (N2\N3 vs N0\N1) | 2.959 | 1.355-6.46 | | **0.006** | | | 1.712 | 0.678-4.327 | | 0.255 |
| pTNM Stage (III\IV vs I\II) | 4.474 | 1.684-11.889 | | **0.003** | | | 2.237 | 0.595-8.407 | | 0.233 |

*P* < 0.05 marked in bold font shows statistical significance.
