# Supplementary material for: Comprehensive multi-omics analysis of pyroptosis for optimizing neoadjuvant immunotherapy in patients with gastric cancer
Source: Theranostics. 2024 May 5;14(7):2915–33. doi: 10.7150/thno.93124 (PMC11103507; doi:10.7150/thno.93124)
Supplement: Supplementary file 1 — Supplementary figures and tables. [file thnov14p2915s1.zip › Supplementary figures and tables/Table S9.docx]

**Table S9. Cox regression analysis of prognostic factors for prognosis.**

| **Variables** | **Validation-4 Central China Cohort (n=100)** | | | | | |
| --- | --- | --- | --- | --- | --- | --- |
|  | **Univariate analysis** | | | **Multivariate analysis** | | |
|  | **HR 95% CI *P*** | | | **HR 95% CI *P*** | | |
| PRS (high vs <low) | 2.633 | 1.372-5.054 | **0.004** | 2.273 | 1.174-4.398 | **0.015** |
| Age (≥65 vs <65) | 2.237 | 1.177-4.252 | 0.14 |  |  |  |
| Gender (male vs female) | 1.743 | 0.775-3.92 | 0.179 |  |  |  |
| BMI (≥25 vs <25) | 0.601 | 0.268-1.352 | 0.219 |  |  |  |
| pT Stage (T3\T4 vs T1\T2) | 3.539 | 1.094-11.451 | **0.035** | 1.221 | 0.211-7.06 | 0.824 |
| pN Stage (N2\N3 vs N0\N1) | 3.559 | 1.931-6.56 | **<0.001** | 2.546 | 1.314-4.933 | **0.006** |
| pTNM Stage (III\IV vs I\II) | 4.237 | 1.665-10.781 | **0.002** | 2.124 | 0.493-9.152 | 0.312 |
| Tumor Size (≥5mm vs <5mm) | 1.26 | 0.677-2.343 | 0.466 |  |  |  |
| CA199 (elevated vs normal) | 1.592 | 0.707-3.586 | 0.262 |  |  |  |
| CEA (elevated vs normal) | 1.308 | 0.606-2.821 | 0.494 |  |  |  |

*P* < 0.05 marked in bold font shows statistical significance.
